# Predicting the Impact of Climate Change on *Corylus* Species Distribution in China: Integrating Climatic, Topographic, and Anthropogenic Factors

**DOI:** 10.1002/ece3.70528

**Published:** 2024-11-03

**Authors:** Yu Liu, Lin Chen

**Affiliations:** ^1^ College of Horticulture and Forestry Sciences/Hubei Engineering Technology Research Center for Forestry Information Huazhong Agricultural University Wuhan China; ^2^ Qinghai Academy of Agriculture and Forestry Qinghai University Xining China

**Keywords:** climate change, conservation strategies, *Corylus* species, ecological model, habitat distribution, human footprint, SSP scenarios

## Abstract

This study investigates the impact of climate change on the distribution of *Corylus* species in China using the MaxEnt model. Key environmental variables, such as Bio6 (mean temperature of the coldest month) and human footprint, emerged as significant determinants of habitat suitability. The study reveals substantial shifts in suitable habitats due to global warming and increased precipitation, with notable expansion towards higher latitudes. Species like *Corylus heterophylla* Fisch. ex Bess. and *Corylus mandshurica* Maxim. demonstrate resilience in extreme conditions, highlighting the importance of specific ecological traits for conservation. Future projections under various SSP scenarios predict continued habitat expansion, emphasizing the need for targeted conservation strategies to address the critical role of human activities. This research highlights the complex interplay between climatic, topographic, and anthropogenic factors in shaping *Corylus* habitats, advocating for integrated adaptive management approaches to ensure their sustainability amid ongoing climate change.

## Graphical abstract

Analysis of climatic, topographic, and anthropogenic factors influencing *Corylus* species distribution. Predictive modeling under various SSP scenarios, indicating continued habitat expansion towards higher latitudes. Identification of critical conservation strategies to address the role of human activities and climatic changes.

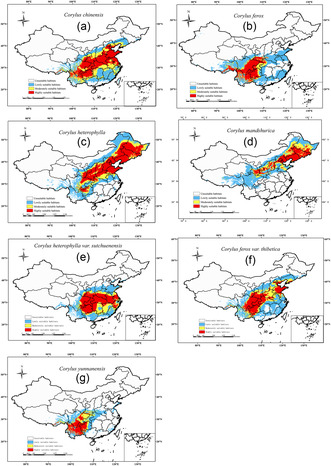

## 1 Introduction

Global climate warming is a well‐documented phenomenon, with each of the past four decades being successively warmer than any preceding decade since 1850 (Quante 2010). Over the past 20 years, China has recorded its highest average temperatures since 1901, leading to significant climatic shifts such as increased rainfall in the north and stable or reduced rainfall in the south, expanding the northern rain belt (Ren et al. 2021). These changes are not only altering weather patterns but also profoundly impacting ecosystems and biodiversity (Mooney et al. 2009; Muluneh 2021). Plant species distributions are particularly sensitive to such changes, with climate acting as a primary driver of geographical spread and regional biodiversity (Thuiller et al. 2013; Coelho et al. 2023). In addition to climate, other factors such as soil type, terrain, and human activities also shape species distributions (Mod et al. 2016; Bradie and Leung 2017). Although numerous studies have focused on the impact of climate change on species distribution (Testolin et al. 2021; Briscoe et al. 2023), limited attention has been paid to the *Corylus* species, particularly in China, where these species contribute both economically and ecologically. Understanding the specific factors influencing *Corylus* distribution in response to climate change remains an understudied area, particularly regarding the integration of climatic, topographic, and anthropogenic factors in distribution modeling.

*Corylus*, one of the world's four major nut‐producing genera (alongside Walnut, Almond, and Cashew Nut), holds significant importance in international trade due to its economic and nutritional value (Allegrini et al. 2022; Lovell et al. 2023 ). In China, *Corylus* is not only an important grain and oil resource but also valued for its cold resistance and medicinal properties, providing substantial ecological benefits such as preventing soil erosion and stabilizing landscapes (Botta et al. 2019; Likhanov et al. 2022; Sun et al. 2024). Globally, 25 species of *Corylus* are distributed across Asia, Europe, and North America, with eight species found in China, primarily in mountainous regions (Mehlenbacher 1991; Holstein, Tamer, and Weigend 2018). As climate change intensifies, understanding the distribution shifts of *Corylus* species in response to these changing environmental conditions is crucial for conservation and sustainable management strategies. Assessing the potential shifts in *Corylus* distribution under future climatic conditions is essential not only for the conservation of *Corylus* species but also for informing broader ecosystem management and adaptation strategies in the face of ongoing environmental challenges (Dawson et al. 2011).

Currently, an increasing number of studies have utilized species distribution models (SDMs) to predict the potential habitats of economically and ecologically important species (Khan et al. 2022; Nguyen and Leung 2022; Yang et al. 2023). SDMs integrate species occurrence records and environmental predictor variables to model the potential distribution of species under various climate scenarios (Milanesi, Della Rocca, and Robinson 2020; Santini et al. 2021). The application of SDMs originated from early studies exploring the relationship between species distributions and environmental gradients (Elith and Leathwick 2009; Whittaker 1956). With advancements in computational tools and statistical methods, various SDMs have been developed, such as generalized linear models (GLM) (Guisan, Edwards, and Hastie 2002), random forest (RF) (Rigatti 2017), and maximum entropy (MaxEnt) (Phillips, Anderson, and Schapire 2006). Among these, the MaxEnt model is widely used due to its ability to work with presence‐only data and its robustness with small sample sizes (Wisz et al. 2008; Kramer‐Schadt et al. 2013; Galante et al. 2018). The reliability of the MaxEnt model in predicting potential habitats of tree species has been well‐documented (Abolmaali, Tarkesh, and Bashari 2018; Kaky et al. 2020; Zhao et al. 2021; Zhao, Wang, and Chen 2024; Liu et al. 2022). Despite the widespread use of SDMs, limited attention has been given to the *Corylus* species, especially within the context of China's diverse climatic and geographic conditions.

Previous research has demonstrated the importance of environmental factors, such as temperature and precipitation, in shaping the geographical ranges of tree species (Li et al. 2020; Ouyang et al. 2021; Huang et al. 2021; De Frenne et al. 2021; Thakur et al. 2022). However, few have comprehensively integrated climatic, topographic, and anthropogenic factors in predicting *Corylus* species distribution, making this study critical for filling knowledge gaps regarding *Corylus* distribution and germplasm conservation under changing climate scenarios. This research hypothesizes that climatic variables, along with topographic and anthropogenic factors, significantly influence *Corylus* habitat suitability.

To identify key environmental factors influencing *Corylus* species distribution, we utilized the MaxEnt model to predict future habitat suitability under various climate scenarios. By integrating species occurrence data with environmental variables, we modeled the potential distribution patterns of *Corylus* species across China. Additionally, we analyzed the implications of these distribution patterns for the conservation of *Corylus* germplasm diversity, focusing on areas suitable for germplasm introduction and breeding. This research aims to provide critical insights into adaptive management strategies that can enhance the resilience of *Corylus* species in the face of climate change, thereby informing conservation practices for long‐term ecosystem sustainability.

## 2 Materials and Methods

### 2.1 Distribution Data

All distribution data were downloaded from the China Virtual Herbarium (CVH) website (http://www.cvh.ac.cn) (accessed on December 23, 2022). Duplicated and ambiguous location sites were deleted. Ambiguous sites refer to those with incomplete or unclear location information, such as missing coordinates. Due to the sensitivity requirements of the MaxEnt model, species with fewer than 30 recorded sites were excluded from the analysis (Gebrewahid et al. 2020). To minimize the effects of spatial autocorrelation and uneven sampling intensity, we selected only one occurrence record per 10 km × 10 km grid cell (Dormann et al. 2007; Merow, Smith, and Silander 2013; Radosavljevic and Anderson 2014; Yang et al. 2023). Ultimately, 1075 occurrence records from 18 *Corylus* species were retained for modeling (Table 1 and Table S1).

**TABLE 1:** The number of records used for model of selected 18 *Corylus* species in this study.

Number	Species	Number of records
1	*Corylus heterophylla* Fisch. ex Bess.	344
2	*Corylus mandshurica* Maxim.	183
3	*Corylus heterophylla* Fisch. ex Bess. var. *sutchuenensis* (Franch.) Rehder.	172
4	*Corylus ferox* Wall.	116
5	*Corylus ferox* Wall. var. *thibetica* (Batalin) Batalin.	97
6	*Corylus yunnanensis* Franch.	88
7	*Corylus chinensis* Franch.	72
8	*Corylus fargesii* Franch.	10
9	*Corylus kweichowensis* Hu var. *brevipes* Franch.	7
10	*Corylus sieboldiana* Blume var. *mandshurica* (Maxim.) Nakai.	6
11	*Corylus mandshurica* Maxim. f. *glandulosa* Franch.	5
12	*Corylus sieboldiana* Blume.	5
13	*Corylus heterophylla* Fisch. ex Bess. var. *brevipes* Rehder.	3
14	*Corylus heterophylla* Fisch. ex Bess. var. *shenyangensis* Rehder.	2
15	*Corylus wangii* Hu.	2
16	*Corylus fargesii* Franch. var. *latifolia* Rehder.	1
17	*Corylus chinensis* Franch. var. *fargesii* Rehder.	1
18	*Corylus heterophylla* Fisch. ex Bess. f. *glandulosa* Franch.	1

### 2.2 Predictor Variables

We selected 44 predictor variables to identify the key factors influencing the distribution patterns of the *Corylus* genus (known as hazel) in China. The bioclimatic variables (bio1–19), wind speed, water vapor pressure, solar radiation, precipitation, mean maximum, and minimum temperatures were obtained directly from the WorldClim version 2.1 dataset (accessed on February 18, 2023), which provides mean values for the period 1970–2000 with a spatial resolution of 30 arcsec (1 km^2^). The accuracy of these data was assessed by means of global correlation coefficients. Correlation coefficients exceeded 0.99 for all temperature parameters, 0.95 for solar radiation and vapor pressure, 0.86 for precipitation variables, and 0.76 for wind speed. The WorldClim 2.1 dataset (Fick and Hijmans 2017) has been referenced in this study. We used a soil dataset (v1.2) based on the World Soil Database (HWSD) (Wieder 2014) (accessed on February 18, 2023), which includes eight attributes for soil type, effective soil water content, and the soil surface layer (0–30 cm). We extracted slope and aspect values from the European Space Agency's Copernicus Global 30‐m Digital Elevation Model (DEM) (European Space Agency and Airbus 2022) (accessed on February 18, 2023). We obtained 1‐km resolution NDVI data from the MOD13A3 dataset under the MODIS program (accessed on February 18, 2023). Additionally, we obtained 1‐km resolution NDVI data from the MOD17A3H dataset, which we downloaded from the Google Earth Engine (GEE) platform (www.earthengine.google.com). Land cover classification data were downloaded from the Copernicus Climate Change Service (C3S) Climate Data Store (CDS) (https://cds.climate.copernicus.eu) (accessed on February 22, 2023). Human footprint (HF) were downloaded from the Centre for Socio‐Economic Data and Applications website (http://www.ciesin.org/) (accessed on February 22, 2023). The data of population (POP) were downloaded from the Center for International Earth Science Information Network (https://sedac.ciesin.columbia.edu/) (accessed on February 22, 2023). To ensure consistency between datasets (Usery et al. 2004; Mugiyo et al. 2022), all data were resampled to match the resolution of the climate data (30 arc‐seconds) using bilinear interpolation in ArcGIS 10.7 (Redlands, CA: Environmental Systems Research Institute).

Climate data (19 bioclimatic variables) were downloaded from WorldClim included Last interglacial (LIG), Last Glacial Maximum (LGM), Mid Holocene (MH), current (1970–2000), 2050s (2041–2060), and 2070s (2061–2080). For each General Circulation Model (GCM), three climate change scenarios were selected: SSP126 (sustainable development path), SSP245 (intermediate path), and SSP370 (regional competition path). The GCMs, including BCC‐CSM2‐MR, CNRM‐CM6‐1, and MIROC6, were chosen based on their proven accuracy in simulating historical climate data for the study region and their frequent use in species distribution modeling (Yang et al. 2021). The selection of SSPs was based on the scenario framework (O'Neill et al. 2016), which provides different trajectories of greenhouse gas emissions, ranging from low emissions (SSP126) to high emissions (SSP370), enabling a comprehensive assessment of potential future climate impacts on the distribution of *Corylus* species. The reliability and accuracy of GCMs and SSPs in predicting future climate conditions have been well‐documented (Meehl et al. 2007; Hausfather and Peters 2020).

Pearson correlation analysis was used to screen the 44 predictor variables. Only variables with correlation coefficients |*r*| ≤ 0.8 were retained (Dormann et al. 2007). To avoid overfitting of the model due to multicollinearity of variables, the variance inflation factor (VIF) analysis (threshold 10) was used to filter predictor variables (Tavallali, Razavi, and Brady 2017). Finally, 24 predictor variables were left for subsequent modeling (Table 2).

**TABLE 2:** Details of the variables and the parameter used in this study.

Parameter	Definition
Bio1	Annual mean temperature (°C)
Bio2*	Mean diurnal range (mean of monthly [max temp − min temp]) (°C)
Bio3*	Isothermality (Bio2/Bio7) (×100)
Bio4	Temperature seasonality (standard deviation × 100) (°C)
Bio5	Maximum temperature in warmest month (°C)
Bio6*	Minimum temperature in coldest month (°C)
Bio7*	Temperature annual range (Bio5–Bio6) (°C)
Bio8	Mean temperature of wettest quarter (°C)
Bio9	Mean temperature of driest quarter (°C)
Bio10	Mean temperature of warmest quarter (°C)
Bio11	Mean temperature of coldest quarter (°C)
Bio12	Annual precipitation (mm)
Bio13	Precipitation of wettest month (mm)
Bio14	Precipitation of driest month (mm)
Bio15*	Precipitation seasonality (coefficient of variation)
Bio16	Precipitation of wettest quarter (mm)
Bio17*	Precipitation of driest quarter (mm)
Bio18	Precipitation of warmest quarter (mm)
Bio19	Precipitation of coldest quarter (mm)
Wind*	Wind speed (m s^−1^)
Vapr	Water vapor pressure (kPa)
Srad	Solar radiation (kJ m^−2^ day^−1^)
Prec	Precipitation (mm)
Tavg	Average temperature (°C)
Tmax*	Maximum temperature (°C)
Tmin	Minimum temperature (°C)
Alt*	Altitude (m)
Slope*	Extract from DEM (°)
Aspect*	Extract from DEM (°)
T_H_2_O_pH*	Top soil pH (0–30 cm) (−log(H^+^))
Soil_type*	Soil type
Awc_class	Soil effective water content
T_oc*	Soil organic carbon content (% weight)
T_texture*	Top soil texture
T_cec_clay*	Soil cation exchange capacity in clay layers (cmol/kg)
T_usda*	Soil texture classification
T_gravel*	Percentage by volume of crushed stone (%vol)
T_silt*	Clay content (% wt)
T_CaCO_3_*	Carbonate content (% weight)
NDVI*	Normalized difference vegetation index
NPP*	Net primary productivity
LCCS*	Land cover classification system
HF*	Human footprint
POP	Population

*Note:* Variables with * indicated that were selected by Pearson correlation (< 0.8) and the variance inflation factor (VIF) analysis (threshold 10) and used for MaxEnt model.

### 2.3 Model Simulation and Evaluation

We used the maximum entropy (MaxEnt, version 3.4.4) to model habitat suitability for the *Corylus* species. MaxEnt is a widely used machine learning algorithm to model species distribution based on presence‐only data and bioclimatic variables (Kalinski 2019).

We utilized the Kuenm package (https://github.com/marlonecobos/kuenm) to optimize the regularization multiplier and feature class parameters within R version 4.2.1 (https://www.r‐project.org/) software. These two parameters are crucial for constructing the species distribution model using MaxEnt. In the MaxEnt model, background points were randomly selected from the study region to represent the environmental space available for *Corylus* species. A total of 10,000 background points were used, a commonly recommended number in MaxEnt modeling (Merow, Smith, and Silander 2013; Yan et al. 2018; Yoon et al. 2023; Yang et al. 2023; Chen et al. 2024), ensuring that the model effectively contrasts presence locations with the available environmental conditions throughout China. During modeling, 75% of the data served as the training set. We then evaluated 1240 candidate models, encompassing combinations of 40 regularization multiplier settings (ranging from 0.1 to 4 at intervals of 0.1) and 29 feature class combinations. Model selection was based on statistical significance (partial receiver operating characteristic curve, ROC), predictive ability (low‐omission rates), and complexity, in that order. Candidate models were screened to retain those demonstrating statistical significance. Subsequently, the model set was refined omitting variables contributing < 5% of variance wherever possible. The model exhibiting the lowest delta Akaike's information criterion (AIC) values (< 2) was chosen among the significant and low‐omission candidate models (Cobos et al. 2019).

To validate the model's performance, we used both area under the receiver operating characteristic curve (AUC) and a 10‐fold cross‐validation approach. The AUC provides a measure of the model's ability to discriminate between presence and background points, and cross‐validation helps assess the model's stability across multiple data subsets. The model utilized the ROC and the area under the ROC curve (AUC) to assess model accuracy. AUC values range from 0 to 1. The model's performance was classified as failure (AUC = 0.5–0.6), poor (AUC = 0.6–0.7), fair (AUC = 0.7–0.8), good (AUC = 0.8–0.9), and excellent (AUC > 0.9). One advantage of this approach is that it provides a single measure of model performance, independent of any specific threshold selection. The higher the AUC value for given bioclimatic variables, the closer the correlation between the variables and the target specie’ geographic distribution model, and the better its predictive performance (Wei et al. 2018).

### 2.4 Data Analysis

The file output by MaxEnt was converted into a grid file using ArcGIS 10.7. The format conversion tool of ArcGIS was used to convert the data into Raster format so that they can be displayed in ArcGIS. The extract analysis was used to get the existence probability distribution map for each species. The probability values range 0–1. Based on this probability, an area was classified either as unsuitable for *Corylus* species (0–0.10), lowly suitable (0.11–0.25), moderately suitable (0.26–0.50), or highly suitable (0.51–1). The criteria for determining habitat suitability levels are experience and previous studies based (Liu, White, and Newell 2013; Liu, Newell, and White 2016; Yan et al. 2018; Chen et al. 2024).

The grid file added the north arrow and other elements using the Arc toolbox tool. Finally, a distribution map of the species' areas of potential habitat suitability was generated.

The SDM (species distribution modeling) tool in ArcGIS was used to calculate the species habitat resistance model by assigning resistance values to various landscape features (Brown, Bennett, and French 2017), such as land cover, topography, and climate factors. Lower resistance values were assigned to areas that facilitate species movement, while higher values represent barriers to dispersal (Ricca et al. 2018; McCluskey et al. 2022).

To determine the migration routes of *Corylus* species, we employed least‐cost path analysis in ArcGIS (Adriaensen et al. 2003). This method uses resistance surfaces to identify the most efficient dispersal corridors between the species' current and projected future habitats, minimizing the cost of movement. The migration routes were visualized as paths connecting areas of suitable habitat under current conditions to those predicted under future climate scenarios (Liao et al. 2020; Lima, Lenoir, and Hylander 2024).

The evaluation of suitable habitat migration involved comparing current (1970–2000) habitat suitability maps with future projections (2050s and 2070s) based on three SSPs (SSP126, SSP245, and SSP370). The MaxEnt model output was used to generate these suitability maps. ArcGIS was used to calculate the spatial shifts in suitable habitats, measuring the distance and direction of these shifts to assess the impact of climate change on the *Corylus* distribution.

All spatial analyses, including resistance modeling, migration route calculation, and habitat migration evaluation, were performed using ArcGIS 10.7 and R (version 4.2.1). This comprehensive approach allowed us to evaluate the potential shifts in *Corylus* species' habitats due to climate change, informing conservation and management strategies.

## 3 Result

### 3.1 Model Accuracy Evaluation

The settings of regularization multiplier (RM) and feature classes (FC) in the MaxEnt algorithm are used to balance model fitting and complexity and determine the types of constraints allowed in the model. In the mode of optimized setting (RM = 0.1, FC = LQPH), the delta AIC_c_ was 0. The lower the delta AIC_c_, the higher the model accuracy, which indicates that the overfitting degree and complexity of the optimized model have been reduced and the optimized model performs “excellent.” Based on the results of the optimized MaxEnt model, the AUC values of each species were calculated (Figure S1). All AUC values were above 0.980, indicating the good performance and high accuracy of the model.

### 3.2 Key Predictor Variables

**FIGURE 1:**
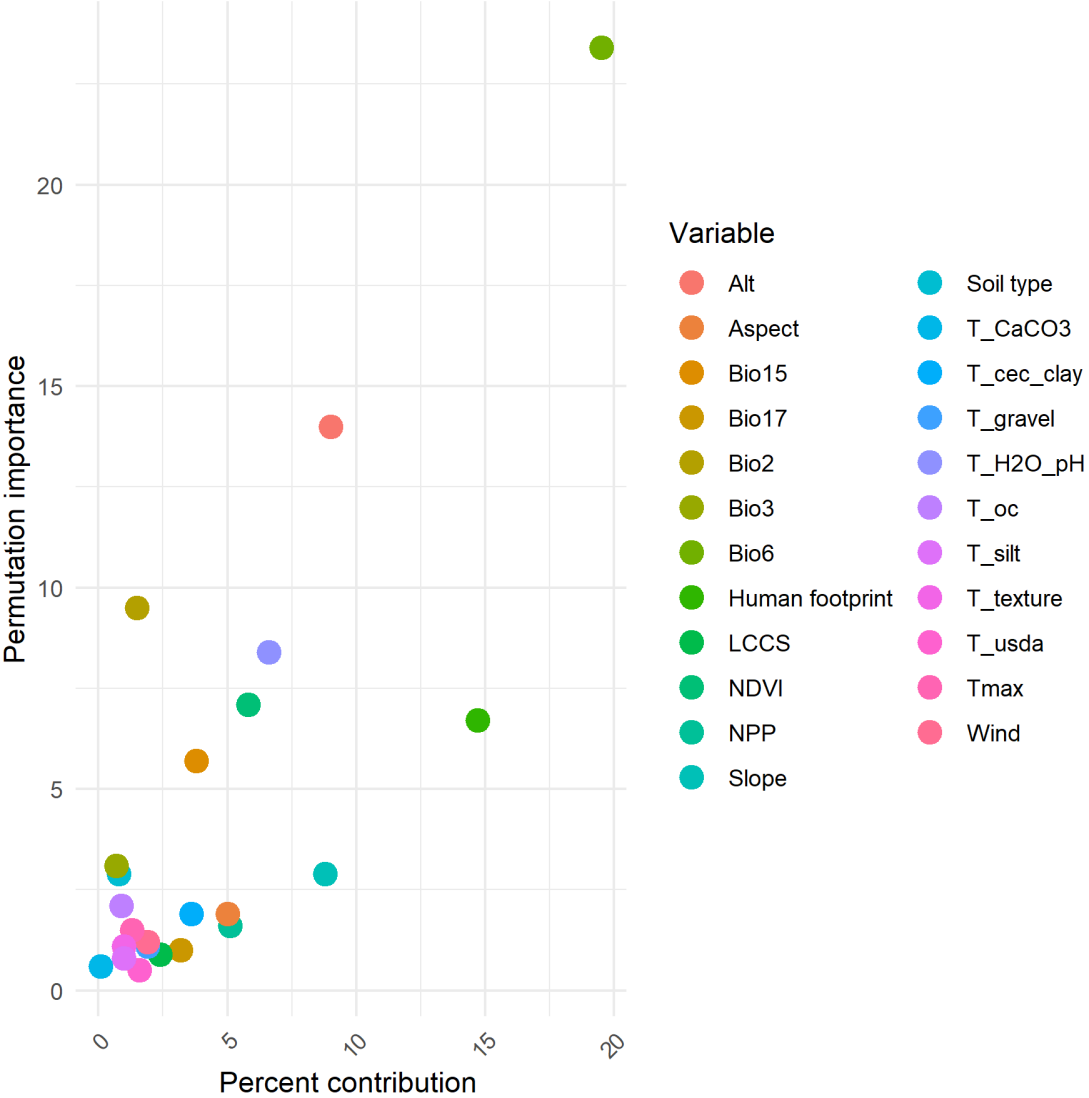
Scatter plot of percent contribution versus permutation importance for various variables.

The MaxEnt model provided the percentage contribution and permutation importance of all predictor variables (Figure 1). In terms of percentage contribution, bioclimatic variables were the main drivers influencing the potential distribution of *Corylus* species in China. Minimum temperature in the coldest month (Bio6) contributed the most to the model. In addition, human footprint (HF), altitude (Alt), and slope (Slope) had high contribution percentages, suggesting that human activities and topographic conditions influenced the distribution of *Corylus* species.

### 3.3 Potential Suitable Habitats of *Corylus* Species in Paleoclimatic

For *C. chinensis* Franch., the suitable habitats accounted for 32.04%, 30.80%, 32.14%, and 35.15% of the national land area during the Last Interglacial (LIG), Last Glacial Maximum (LGM), Mid Holocene (MH), and the current period, respectively. For *C. ferox* Wall., the suitable habitats accounted for 14.97%, 13.49%, 14.41%, and 25.37% of the national land area during the LIG, LGM, MH, and the current period, respectively. For *C. heterophylla*, the suitable habitats accounted for 41.44%, 37.01%, 36.81%, and 41.11% of the national land area during the LIG, LGM, MH, and the current period, respectively. For *C. mandshurica* Maxim., the suitable habitats accounted for 25.32%, 24.14%, 20.74%, and 32.54% of the national land area during the LIG, LGM, MH, and the current period, respectively. For *C. heterophylla* Fisch. ex Bess. var. *sutchuenensis* (Franch.) Rehder., the suitable habitats accounted for 22.37%, 20.71%, 20.92%, and 27.41% of the national land area during the LIG, LGM, MH, and the current period, respectively. For *C. ferox* Wall. var. *thibetica* (Batalin) Batalin., the suitable habitats accounted for 20.12%, 16.48%, 17.95%, and 32.72% of the national land area during the LIG, LGM, MH, and the current period, respectively. For *C. yunnanensis* Franch., the suitable habitats accounted for 9.43%, 10.41%, 10.27%, and 15.40% of the national land area during the LIG, LGM, MH, and the current period, respectively.

### 3.4 Current Potential Distribution

**FIGURE 2:**
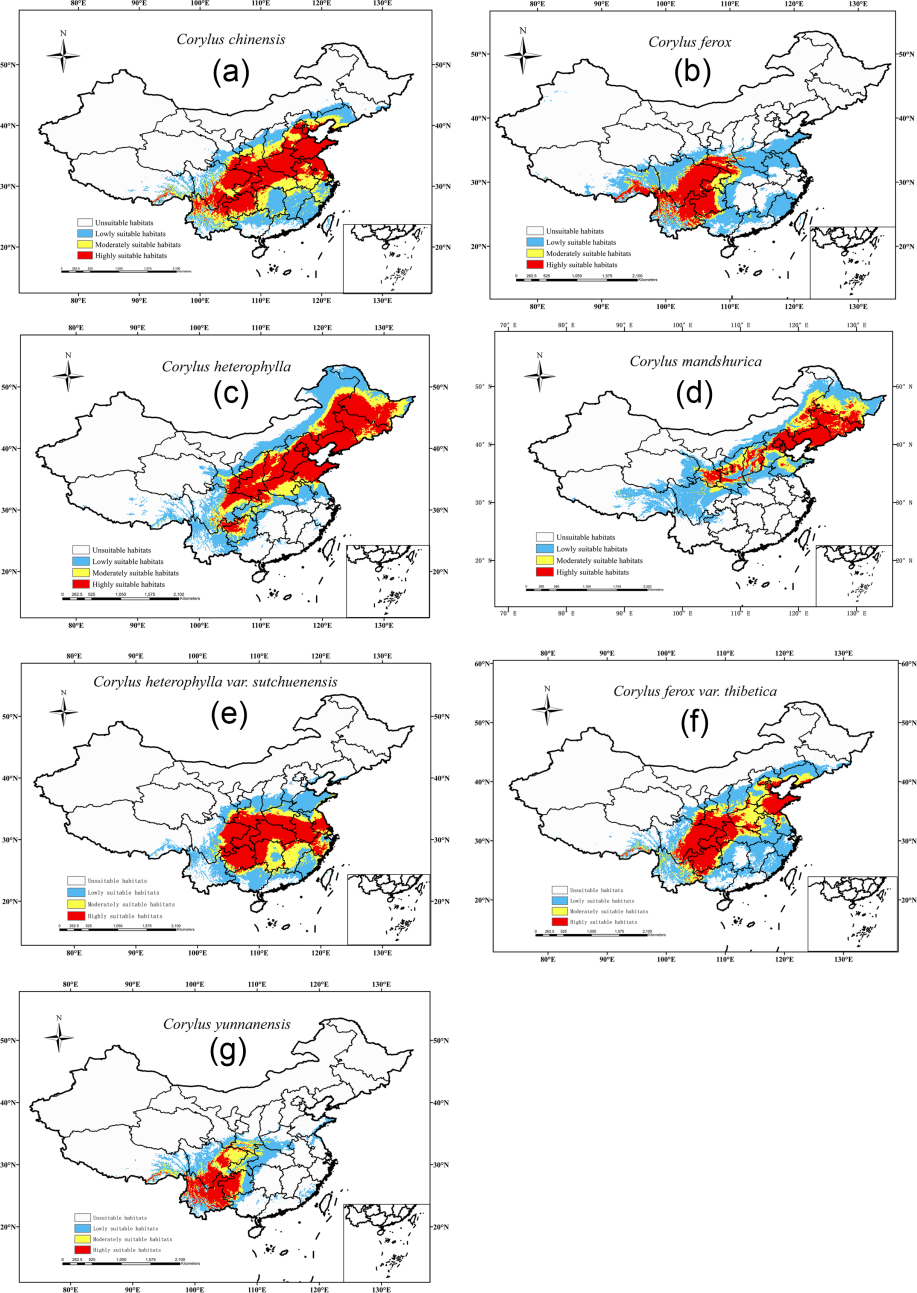
Potential distribution pattern of 7 *Corylus* species under current climate. (a) *C. chinensis*, (b) *C. ferox*, (c) *C. heterophylla*, (d) *C. mandshurica*, (e) *C. heterophylla* var. *sutchuenensis*, (f) *C. ferox* var. *thibetica*, and (g) *C. yunnanensis*.

In the current period, the distribution of suitable habitats for different *Corylus* species varies significantly: the moderately and highly suitable habitats for *C. chinensis* are mainly concentrated in North China and Southwest China (Figure 2a). For *C. ferox*, the suitable habitats are primarily concentrated in Southwest China (Figure 2b). *C. heterophylla* is mainly found in North China, Northeast China, and parts of Southwest China (Figure 2c). *C. mandshurica* is primarily concentrated in Northeast China and parts of North China (Figure 2d). The moderately and highly suitable habitats for *C. heterophylla* var. *sutchuenensis* are mainly located in North China and parts of Southwest China (Figure 2e). *C. ferox* var. *thibetica* is predominantly found in Southwest China and parts of North China (Figure 2f). *C. yunnanensis* is mainly concentrated in parts of Southwest China (Figure 2g).

### 3.5 Future Potential Distribution Under Different Time Periods and Climate Change Scenarios

Compared to the current period, the suitable habitats for *Corylus* species are projected to increase significantly by the 2050s and 2070s under various SSP scenarios. For *C. chinensis*, increases are up to 4.71% in the 2050s and up to 6.03% in the 2070s. For *C. ferox*, increases are up to 32.83% in the 2050s and up to 33.44% in the 2070s. For *C. heterophylla*, increases are up to 25.89% in the 2050s and up to 28.20% in the 2070s. For *C. mandshurica*, increases are up to 10.42% in the 2050s with a slight decrease of 1.93% under SSP370 by the 2070s. For *C. heterophylla* var. *sutchuenensis*, increases are up to 13.34% in the 2050s and up to 16.61% in the 2070s. For *C. ferox* var. *thibetica*, increases are up to 15.56% in the 2050s and up to 15.51% in the 2070s. For *C. yunnanensis*, increases are up to 62.83% in the 2050s and up to 49.75% in the 2070s.

These projections indicate a significant expansion of suitable habitats for most *Corylus* species, suggesting that future climatic conditions will generally favor their growth. This information is critical for developing conservation strategies and optimizing the management and utilization of *Corylus* resources in China (Figure S2).

### 3.6 The Change in the Potential Suitable Habitat of *Corylus* in Different Periods

**FIGURE 3:**
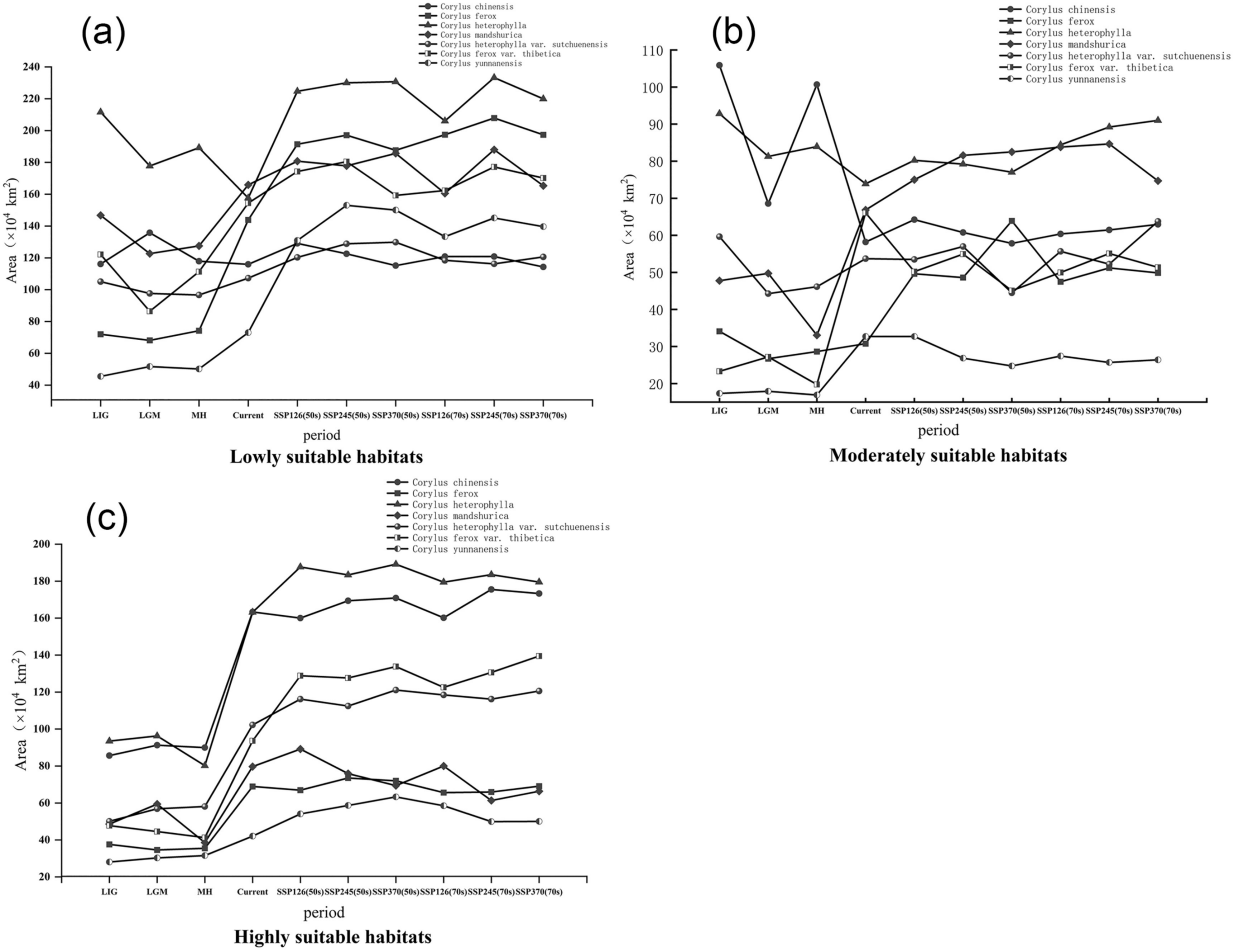
The trend of the area of suitable areas during the same period for 7 *Corylus* species. (a) Changes in the area of low suitability habitats, (b) changes in the area of moderately suitability habitats, (c) changes in the area of highly suitability habitats.

Future projections indicate varied trends for *Corylus* species habitats under different SSP scenarios (Figure 3). For *C. chinensis*, SSP126 shows initial increases in total medium and low suitable areas followed by decreases, while high suitable areas decrease first then increase. SSP245 sees continuous growth in medium, high, and total areas, with low areas first increasing then decreasing. SSP370 predicts continuous decreases in low areas, and increases in high and total areas. For *C. ferox* under SSP126, there are sustained increases in low and total areas, with medium and high areas initially increasing then decreasing. SSP245 sees continuous growth in all areas, while SSP370 predicts sustained increases in low areas and mixed trends in others. For *C. heterophylla*, SSP126 predicts increases in low and high zones, while medium and total zones increase first then decrease. SSP245 sees continuous growth across all zones, and SSP370 predicts decreases in low zones and mixed trends in others. For *C. mandshurica* under SSP126, there is growth in all areas with initial increases in low and high areas. SSP245 predicts continuous increases, while SSP370 sees decreases in low areas and growth in high areas. For *C. heterophylla* var. *sutchuenensis*, SSP126 shows increases in low and high areas initially, followed by decreases, while medium and total areas continuously grow. SSP245 predicts continuous growth in all areas, while SSP370 sees decreases in low areas and mixed trends in others. For *C. ferox* var. *thibetica* under SSP126, there are increases in low, high, and total areas with continuous decreases in medium areas. SSP245 and SSP370 predict mixed trends with significant changes in high and medium areas. For *C. yunnanensis*, SSP126 predicts growth in low and high areas with medium and total areas increasing first then decreasing. SSP245 shows continuous growth, while SSP370 sees decreases in low areas and mixed trends in others. These projections highlight the need for adaptive conservation strategies to manage *Corylus* species under changing climatic conditions.

### 3.7 Migration Route of *Corylus* Species

**FIGURE 4:**
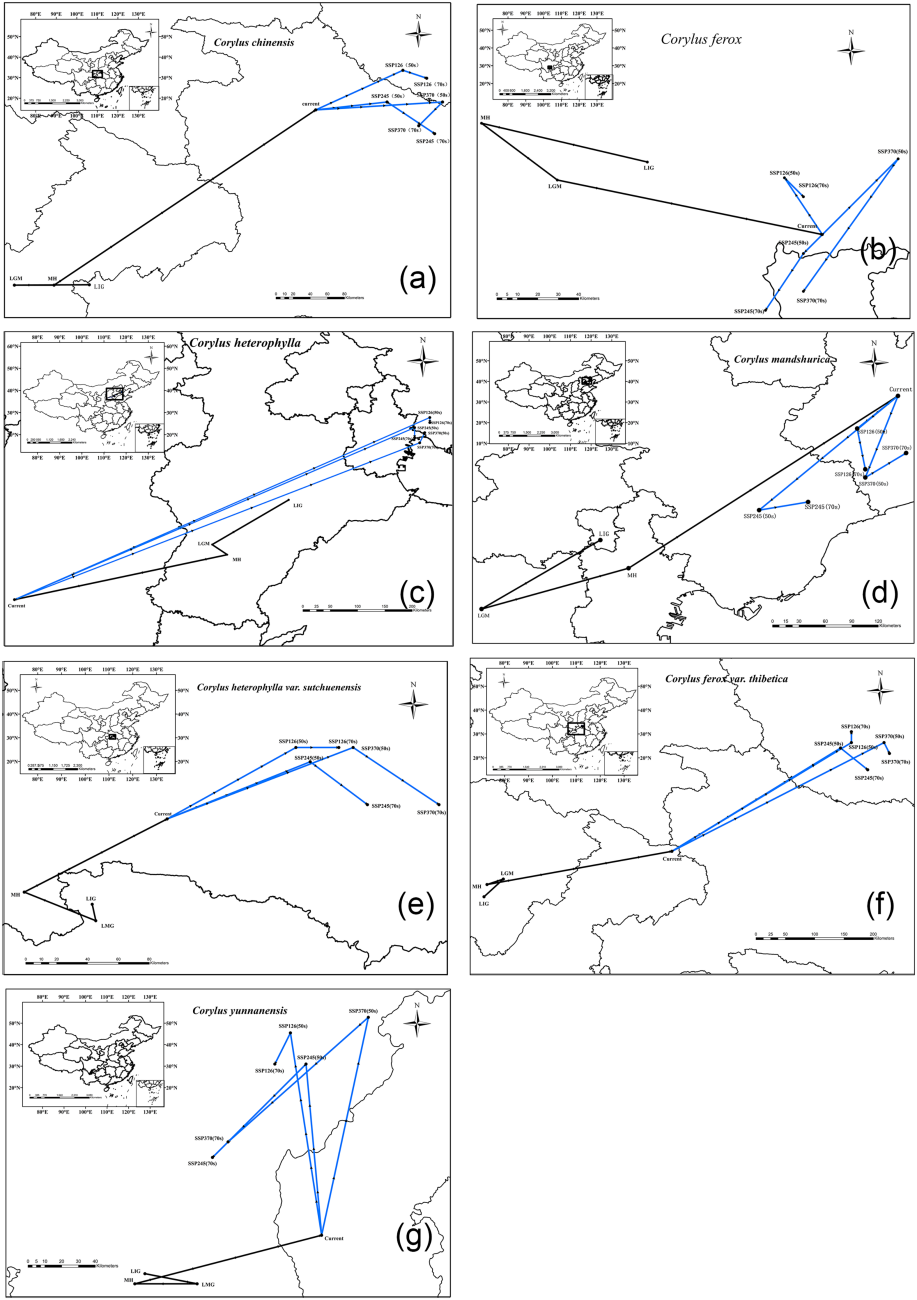
With the climate change, the distribution core changes in 7 *Corylus* species. (a) *C. chinensis*, (b) *C. ferox*, (c) *C. heterophylla*, (d) *C. mandshurica*, (e) *C. heterophylla* var. *sutchuenensis*, (f) *C. ferox* var. *thibetica*, and (g) *C. yunnanensis*. The trend of the blue line represents the change from the current to the next 3 SSP scenarios, and the trend of the black line represents the change from the paleoclimate to the current period.

The distribution cores of *Corylus* species are projected to shift significantly due to climate change (Figure 4). For *C. chinensis*, the core has moved from Hubei Province during the LIG to Chongqing City during the LGM and MH periods, and back to Hubei in the current period. Future projections show a shift to Henan Province by the 2040s–2060s under SSP126, SSP245, and SSP370 scenarios. *C. ferox* has its core in Sichuan Province during the LIG, LGM, and MH periods, and it remains in Sichuan currently, with future shifts predicted within Sichuan under all SSP scenarios. Similarly, *C. heterophylla* shifts from Hebei Province in historical periods to Shanxi Province currently, with future projections indicating movement to Hebei under all SSP scenarios. *C. mandshurica* shows a shift from Hebei and Tianjin in historical periods to Liaoning currently, with future shifts within Liaoning and neighboring areas. *C. heterophylla* var. *sutchuenensis* and *C. ferox* var. *thibetica* display similar patterns of historical stability, with future shifts to neighboring regions under various scenarios. *C. yunnanensis* shifts from Sichuan historically to Yunnan currently, with future movements predicted within Yunnan. These shifts highlight the dynamic nature of *Corylus* species' habitats in response to climate change, underscoring the need for adaptive conservation strategies. The following sections will delve into the environmental factors driving these shifts and their implications for biodiversity and ecosystem management.

### 3.8 Overlapping Areas of Suitable Habitat Distribution for Different Species of *Corylus* Plants in Contemporary

The overlapping distribution areas of *Corylus* species vary across regions and highlight significant biodiversity hotspots. For instance, *C. chinensis* and *C. ferox* overlap mainly in southeastern Sichuan, northeastern Guizhou, northern Chongqing, northern Yunnan, and southern Shaanxi. *C. chinensis* and *C. heterophylla* share areas in northern Guizhou, northeastern Sichuan, southern Shaanxi, northern Hubei, northern Henan, southern Gansu, southern Hebei, Beijing, Tianjin, and Shandong. Additionally, *C. chinensis*, *C. ferox*, and *C. heterophylla* overlap in northeastern Sichuan and northwestern Guizhou. In contrast, *C. chinensis* and *C. mandshurica* have limited overlap mainly in Beijing, Tianjin, southern Gansu, southern Shaanxi, central Shandong, and the Shanxi‐Hebei border.

The distribution cores show significant shifts due to climate change. For example, *C. chinensis* has moved from Hubei during the LIG to Chongqing during the LGM and MH periods, and back to Hubei currently, with future shifts to Henan predicted under various SSP scenarios. *C. ferox* has its core in Sichuan historically and currently, with future shifts within the province expected. Similar patterns are observed for other *Corylus* species, indicating dynamic habitat changes in response to climate conditions. These projections underscore the need for adaptive conservation strategies to manage *Corylus* species' habitats effectively. The following sections will delve into the environmental factors driving these distributions and their implications for biodiversity and ecosystem management (Figure S3).

## 4 Discussion

Our analysis identifies Bio6 (mean temperature of the coldest month, 19.5%) and human footprint (14.7%) as the most significant factors affecting the distribution of *Corylus* species, followed by altitude (9%), slope (8.8%), and soil pH (t_H_2_O_pH, 6.6%) (Figure 1). This demonstrates a complex interplay between climatic, topographic, and anthropogenic factors in shaping the habitats of *Corylus* species.

This study aligns with previous research that underscores the importance of humidity and temperature in the distribution of *Corylus* species (Lenda et al. 2017; Malkiewicz, Drzeniecka‐Osiadacz, and Krynicka 2016; Ustaoglu 2014). Specifically, annual precipitation and average annual temperature have been highlighted as crucial climatic factors (Ustaoglu 2014; Malkiewicz, Drzeniecka‐Osiadacz, and Krynicka 2016). However, our findings bring to light the substantial impact of human activities, emphasizing the need for conservation strategies that integrate both natural and anthropogenic influences.

From the Middle Holocene to the current, the suitable habitats for *Corylus* species have expanded significantly due to global warming and changes in precipitation patterns (Figure 4). The high suitability areas for species like *C. chinensis* and *C. ferox* have increased notably, reflecting their adaptability to warmer and wetter conditions (Figure 3). This expansion is consistent with global trends of rising temperatures and increased humidity, particularly in inland China due to enhanced westerly circulation (Figure 4 and Figure S2).

The shifts in distribution cores for *Corylus* species further illustrate the dynamic nature of their habitats in response to climate change (Finsinger et al. 2006; Theuerkauf et al. 2014; Seppä et al. 2015). For instance, *C. chinensis* has seen its core move from Hubei to Chongqing and back, with future projections indicating a shift towards Henan under various SSP scenarios. Such movements suggest that *Corylus* species are continuously seeking optimal growing conditions as climatic variables change (Figure 4).

Our findings also highlight the resilience of certain species in extreme conditions. For example, regions with low precipitation and temperatures, such as northwest China, remain unsuitable for species like *C. chinensis*, *C. ferox*, and *C. yunnanensis*. However, *C. heterophylla* and *C. mandshurica* thrive in these regions due to their robust cold resistance and adaptability to various soil types (Ma et al. 2023). This underscores the importance of considering specific ecological traits when developing conservation and breeding programs.

Future projections under different SSP scenarios suggest continued habitat expansion for *Corylus* species, particularly towards higher latitudes (Figure S2). The inherent adaptability of these species, characterized by their tolerance to drought, shade, heat, and cold, supports this trend (Andersen 1994; Catoni et al. 2015). For instance, *C. heterophylla* demonstrates remarkable resilience, tolerating extreme cold and thriving in a wide pH range, ensuring its continued habitat expansion even as climate conditions change (Figure 2 and Figure S2).

The overlapping distribution areas of *Corylus* species reveal important biodiversity hotspots. In the 2050s and 2070s, overlaps between *C. chinensis* and other species like *C. ferox* and *C. heterophylla* are expected to increase under SSP126 and SSP370, while overlaps with *C. mandshurica* are projected to decrease (Figure S3). These patterns highlight the need for targeted conservation efforts in regions of high overlap to maintain ecological balance and biodiversity.

Moreover, the study emphasizes the need for a holistic approach to conservation that integrates multiple environmental variables (Table 2). While the MaxEnt model provides a robust framework for predicting suitable habitats, it is crucial to incorporate additional factors such as soil conditions, human activities, and interspecies relationships (Ye et al. 2022). These variables, although challenging to quantify, play a significant role in determining the actual distribution and viability of species (Buckley et al. 2010; Guillera‐Arroita 2017; Guisan et al. 2013).

The role of human footprint as a significant factor in *Corylus* distribution cannot be understated (Figure 1). As human activities continue to alter natural landscapes, it becomes imperative to develop conservation strategies that mitigate these impacts (Scherr and McNeely 2008; Leu, Hanser, and Knick 2008; Reid et al. 2019). This includes protecting critical habitats, promoting sustainable land use practices, and engaging local communities in conservation efforts.

In summary, this study provides a comprehensive analysis of the factors influencing *Corylus* distribution and highlights the significant impact of climate change and human activities. The findings underscore the necessity for adaptive conservation strategies that integrate natural and anthropogenic factors to ensure the resilience and sustainability of *Corylus* species. Future research should aim to refine these models by incorporating a broader range of environmental variables and exploring the synergistic effects of climate change and human activities. Such an approach will be crucial for maintaining the ecological integrity and biodiversity of *Corylus* habitats in the face of ongoing climatic changes.

## 5 Conclusions

This study highlights the significant influence of climatic and anthropogenic factors on the distribution of *Corylus* species in China, with Bio6 (mean temperature of the coldest month) and human footprint emerging as critical determinants. The findings underscore the necessity of integrating natural and human‐induced factors into conservation strategies. Notably, the suitable habitats for *Corylus* species have expanded significantly from the Middle Holocene to the present, driven by global warming and increased precipitation. Geographical shifts in distribution cores, particularly towards higher latitudes, emphasize the need for adaptive management strategies. The resilience of species like *C. heterophylla* and *C. mandshurica* in harsh environments highlights the importance of considering specific ecological traits in conservation programs. Future projections under various SSP scenarios suggest continued habitat expansion, although human activities remain a significant concern. Overlapping distribution areas identify key biodiversity hotspots requiring targeted conservation efforts. In summary, this study underscores the complex interplay of climatic, topographic, and anthropogenic factors in shaping *Corylus* habitats and calls for integrated strategies to ensure their resilience and sustainability in the face of ongoing climate change.

## Author Contributions

**Yu Liu:** conceptualization (equal), data curation (equal), formal analysis (equal), investigation (equal), methodology (equal), software (equal), writing – original draft (equal). **Lin Chen:** conceptualization (equal), funding acquisition (equal), investigation (equal), methodology (equal), resources (equal), supervision (equal), writing – review and editing (equal).

## Conflicts of Interest

The authors declare no conflicts of interest.

## Supporting information


Appendix S1.

## Data Availability

All data are in the main text or the Appendix S1.
