# Supplementary material for: Predicting the Impact of Climate Change on Corylus Species Distribution in China: Integrating Climatic, Topographic, and Anthropogenic Factors
Source: Ecol Evol. 2024 Nov 3;14(11):e70528. doi: 10.1002/ece3.70528 (PMC11532234; doi:10.1002/ece3.70528)
Supplement: Supplementary file 1 — Appendix S1. [file ECE3-14-e70528-s001.zip › ece370528-sup-0001-AppendixS1.docx]

**Supplementary Information**


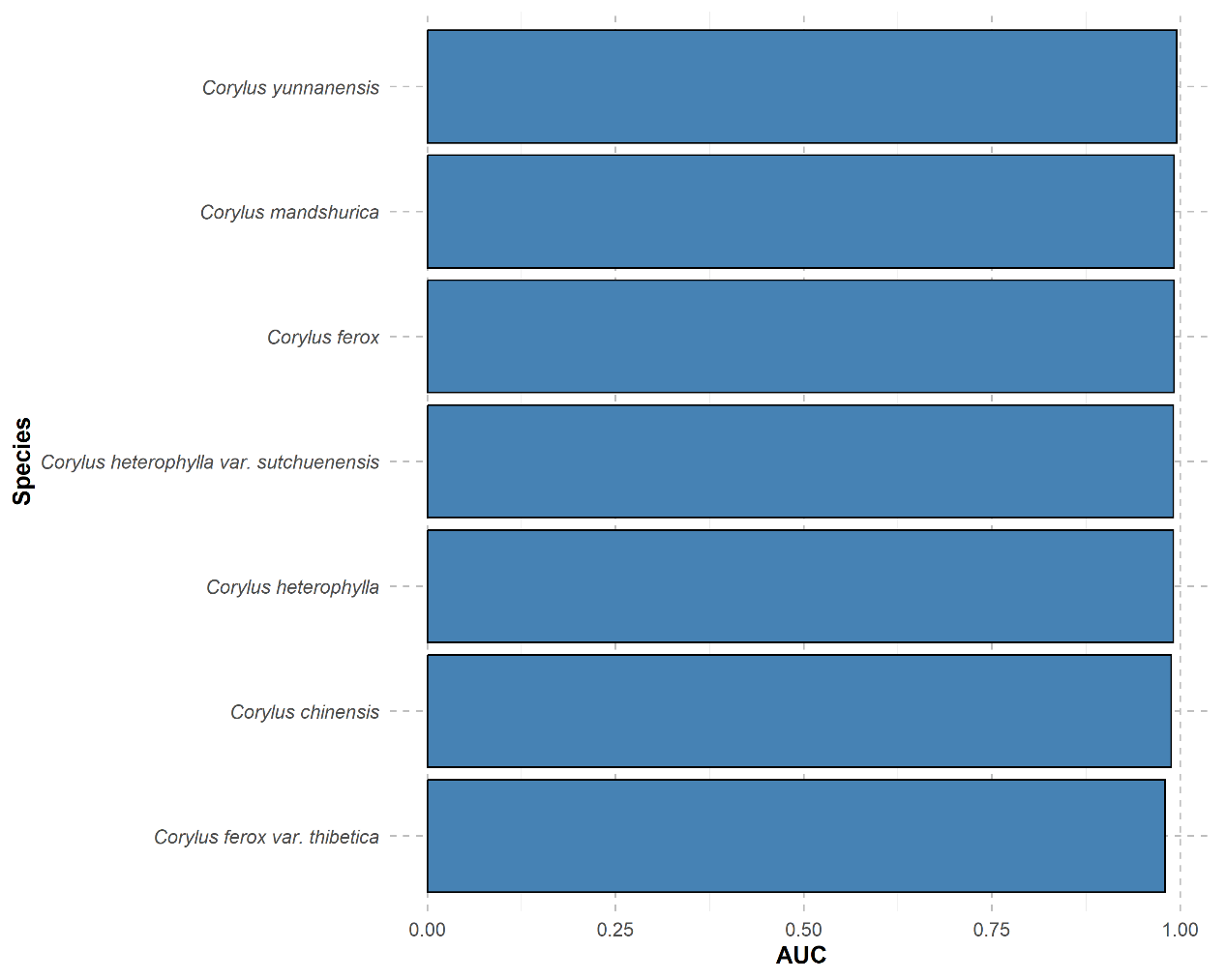


**Fig. S1** The AUC values of 7 *Corylus* species.


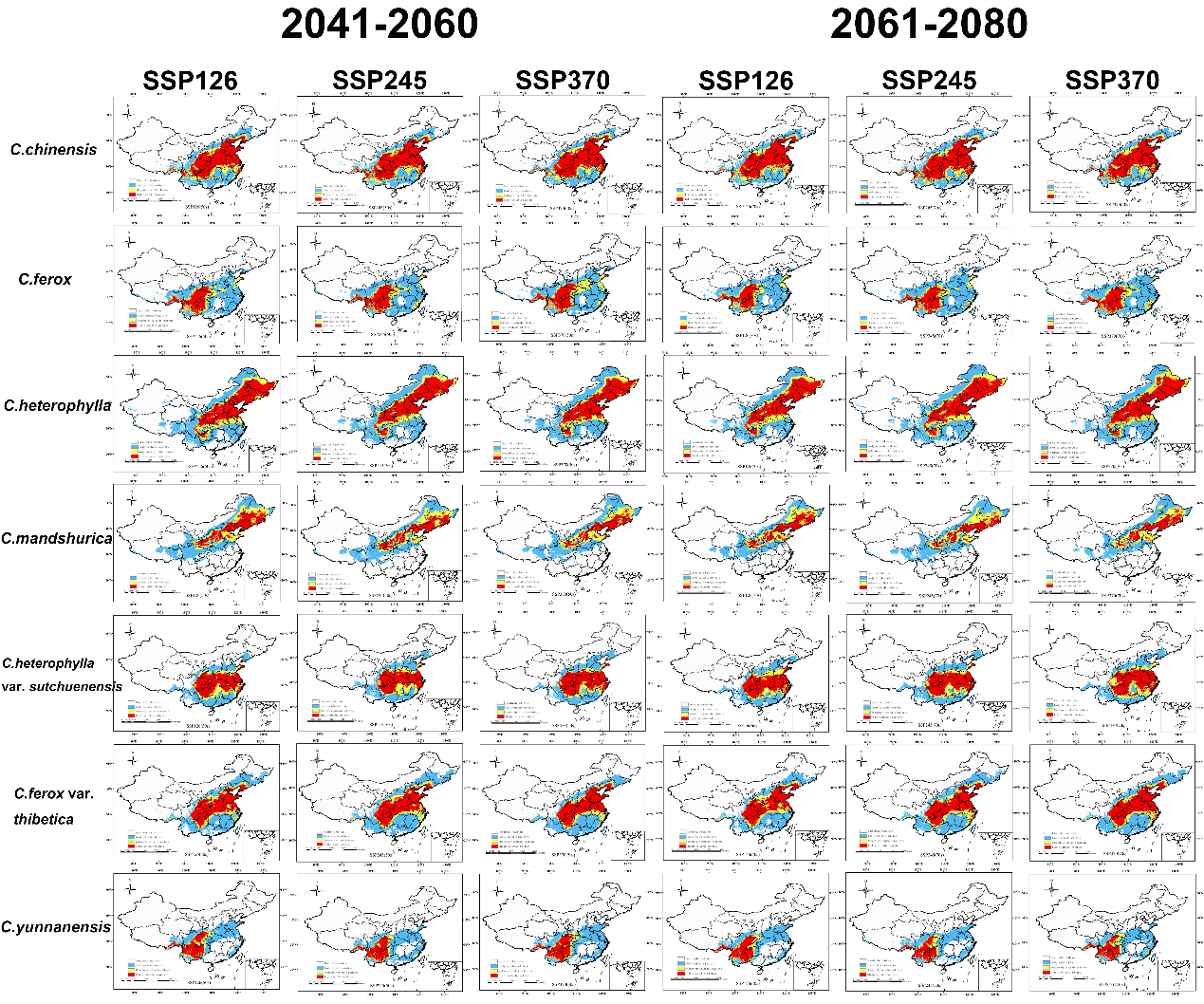


**Fig. S2** Distribution of suitable areas of *Corylus* species under future climate condition


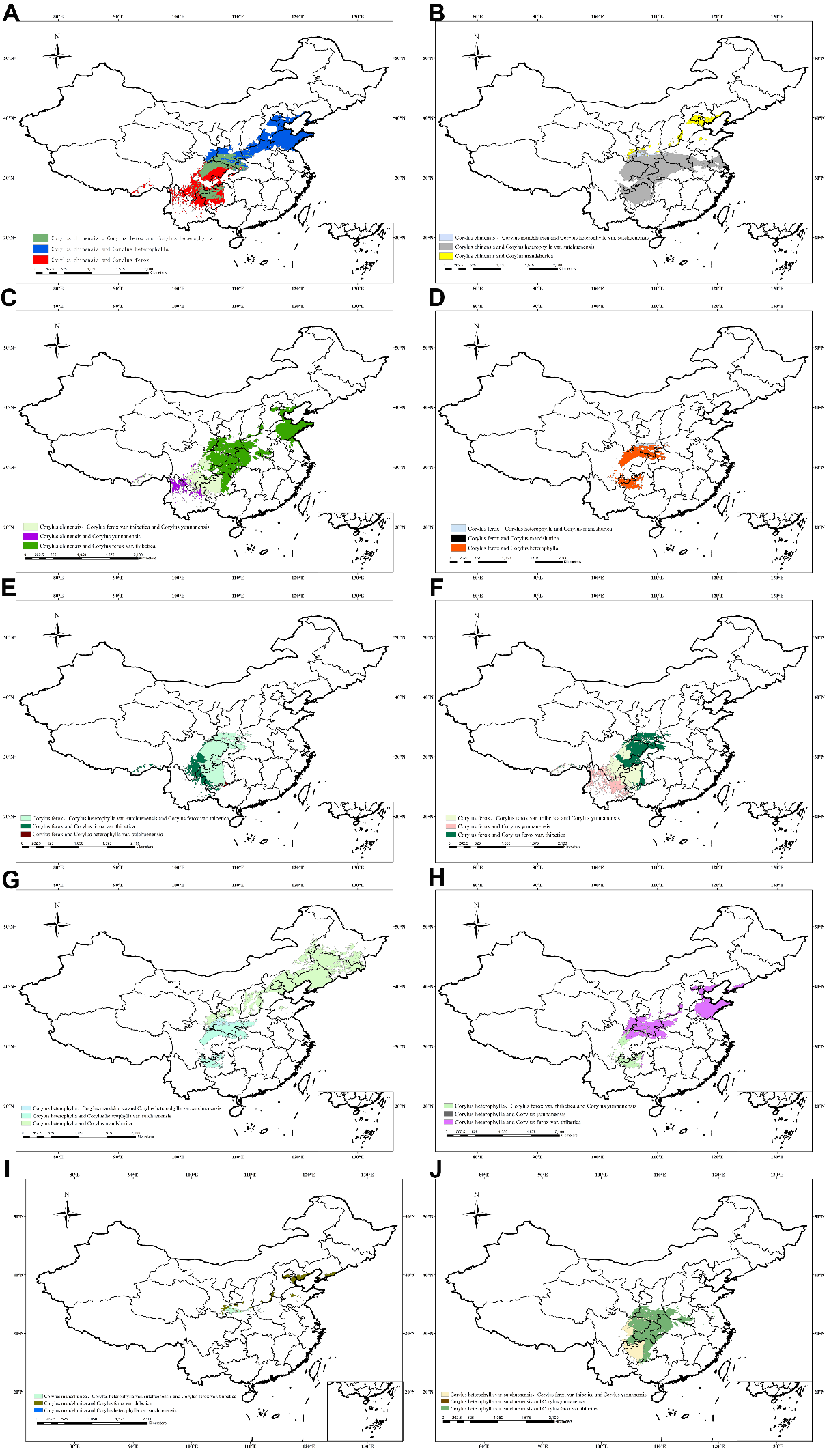


**Fig. S3** The overlapping areas of current distribution for different *Corylus* species.

**Table S1.** Species distribution latitude and longitude points for MaxEnt modelling.

| Species | longtitude | latitude |
| --- | --- | --- |
| *Corylus chinensis* | 109.751589 | 38.234416 |
| *Corylus chinensis* | 104.074176 | 35.804469 |
| *Corylus chinensis* | 110.931342 | 31.072304 |
| *Corylus chinensis* | 104.246756 | 33.266752 |
| *Corylus chinensis* | 98.924453 | 28.470086 |
| *Corylus chinensis* | 100.213525 | 27.33206 |
| *Corylus chinensis* | 100.182492 | 26.566197 |
| *Corylus chinensis* | 100.179764 | 26.257281 |
| *Corylus chinensis* | 99.928328 | 26.99412 |
| *Corylus chinensis* | 100.223139 | 26.893324 |
| *Corylus chinensis* | 102.4258 | 25.2358 |
| *Corylus chinensis* | 103.4258 | 26.2358 |
| *Corylus chinensis* | 103.043564 | 25.344409 |
| *Corylus chinensis* | 99.293688 | 27.191496 |
| *Corylus chinensis* | 99.293466 | 27.18317 |
| *Corylus chinensis* | 103.23434 | 25.238506 |
| *Corylus chinensis* | 108.416592 | 27.999823 |
| *Corylus chinensis* | 104.76979 | 34.531643 |
| *Corylus chinensis* | 118.345723 | 29.723896 |
| *Corylus chinensis* | 104.555635 | 33.896307 |
| *Corylus chinensis* | 116.9516 | 36.633805 |
| *Corylus chinensis* | 106.816059 | 26.64538 |
| *Corylus chinensis* | 105.653149 | 25.097851 |
| *Corylus chinensis* | 110.884272 | 33.783801 |
| *Corylus chinensis* | 114.083414 | 31.81925 |
| *Corylus chinensis* | 111.15343 | 31.646964 |
| *Corylus chinensis* | 114.411481 | 30.549513 |
| *Corylus chinensis* | 110.345247 | 31.434348 |
| *Corylus chinensis* | 110.282825 | 31.673903 |
| *Corylus chinensis* | 111.0599 | 30.71659 |
| *Corylus chinensis* | 125.334427 | 43.895371 |
| *Corylus chinensis* | 112.684989 | 27.280462 |
| *Corylus chinensis* | 112.793142 | 26.999968 |
| *Corylus chinensis* | 110.582579 | 27.065975 |
| *Corylus chinensis* | 114.119248 | 28.417509 |
| *Corylus chinensis* | 110.186181 | 29.373772 |
| *Corylus chinensis* | 110.170413 | 29.405923 |
| *Corylus chinensis* | 110.100227 | 26.413673 |
| *Corylus chinensis* | 109.19492 | 28.412868 |
| *Corylus chinensis* | 111.019245 | 26.447665 |
| *Corylus chinensis* | 110.476145 | 29.359711 |
| *Corylus chinensis* | 120.764344 | 31.070932 |
| *Corylus chinensis* | 114.979679 | 28.99257 |
| *Corylus chinensis* | 117.228124 | 36.364409 |
| *Corylus chinensis* | 120.185719 | 36.411993 |
| *Corylus chinensis* | 107.340613 | 33.162399 |
| *Corylus chinensis* | 106.854547 | 34.582467 |
| *Corylus chinensis* | 114.4843 | 37.14904 |
| *Corylus chinensis* | 115.4843 | 38.14904 |
| *Corylus chinensis* | 116.4843 | 39.14904 |
| *Corylus chinensis* | 106.877362 | 34.247693 |
| *Corylus chinensis* | 108.381363 | 32.984914 |
| *Corylus chinensis* | 109.026785 | 32.216875 |
| *Corylus chinensis* | 108.499139 | 33.462799 |
| *Corylus chinensis* | 109.28277 | 32.18761 |
| *Corylus chinensis* | 110.28277 | 33.18761 |
| *Corylus chinensis* | 111.28277 | 34.18761 |
| *Corylus chinensis* | 112.28277 | 35.18761 |
| *Corylus chinensis* | 113.28277 | 36.18761 |
| *Corylus chinensis* | 114.28277 | 37.18761 |
| *Corylus chinensis* | 109.8015 | 31.58607 |
| *Corylus chinensis* | 110.8015 | 32.58607 |
| *Corylus chinensis* | 111.8015 | 33.58607 |
| *Corylus chinensis* | 110.040894 | 36.697183 |
| *Corylus chinensis* | 107.902133 | 34.128643 |
| *Corylus chinensis* | 103.558245 | 30.856565 |
| *Corylus chinensis* | 103.058726 | 28.859708 |
| *Corylus chinensis* | 103.618516 | 28.518353 |
| *Corylus chinensis* | 105.450108 | 30.150767 |
| *Corylus chinensis* | 98.873475 | 28.300246 |
| *Corylus chinensis* | 109.013574 | 28.453448 |
| *Corylus chinensis* | 108.774586 | 28.84704 |
| *Corylus ferox* | 107.00115 | 34.223102 |
| *Corylus ferox* | 106.461499 | 29.377239 |
| *Corylus ferox* | 103.887469 | 29.241862 |
| *Corylus ferox* | 105.613576 | 31.319302 |
| *Corylus ferox* | 103.352736 | 29.561441 |
| *Corylus ferox* | 103.5652 | 27.2303 |
| *Corylus ferox* | 104.5652 | 28.2303 |
| *Corylus ferox* | 111.919478 | 33.51763 |
| *Corylus ferox* | 111.099086 | 31.71861 |
| *Corylus ferox* | 107.763461 | 34.170077 |
| *Corylus ferox* | 103.356304 | 29.573827 |
| *Corylus ferox* | 103.34249 | 31.07511 |
| *Corylus ferox* | 102.647628 | 30.457022 |
| *Corylus ferox* | 102.703259 | 30.672286 |
| *Corylus ferox* | 102.818478 | 27.712129 |
| *Corylus ferox* | 108.671612 | 31.953391 |
| *Corylus ferox* | 103.193677 | 30.792419 |
| *Corylus ferox* | 103.13238 | 30.463363 |
| *Corylus ferox* | 103.133831 | 30.473462 |
| *Corylus ferox* | 103.350642 | 29.55094 |
| *Corylus ferox* | 104.090358 | 30.660054 |
| *Corylus ferox* | 103.856585 | 31.232898 |
| *Corylus ferox* | 108.292818 | 31.670932 |
| *Corylus ferox* | 103.385792 | 29.911682 |
| *Corylus ferox* | 101.887279 | 29.599382 |
| *Corylus ferox* | 104.495712 | 30.95402 |
| *Corylus ferox* | 103.334253 | 29.570778 |
| *Corylus ferox* | 102.526935 | 29.093935 |
| *Corylus ferox* | 104.4245 | 32.2958 |
| *Corylus ferox* | 104.4246 | 32.2958 |
| *Corylus ferox* | 114.249932 | 26.971132 |
| *Corylus ferox* | 102.241432 | 29.920402 |
| *Corylus ferox* | 102.065228 | 29.587629 |
| *Corylus ferox* | 101.768185 | 28.451666 |
| *Corylus ferox* | 102.028749 | 29.704917 |
| *Corylus ferox* | 102.250211 | 29.913285 |
| *Corylus ferox* | 102.584462 | 26.640824 |
| *Corylus ferox* | 103.578338 | 28.266627 |
| *Corylus ferox* | 103.363567 | 28.33262 |
| *Corylus ferox* | 103.477009 | 28.944241 |
| *Corylus ferox* | 104.263692 | 32.170762 |
| *Corylus ferox* | 102.012457 | 27.218852 |
| *Corylus ferox* | 103.35228 | 29.557783 |
| *Corylus ferox* | 103.842593 | 31.887657 |
| *Corylus ferox* | 103.138586 | 28.33402 |
| *Corylus ferox* | 103.190714 | 28.766367 |
| *Corylus ferox* | 107.216111 | 29.127205 |
| *Corylus ferox* | 104.534276 | 32.41517 |
| *Corylus ferox* | 104.561927 | 32.078563 |
| *Corylus ferox* | 113.149323 | 24.879286 |
| *Corylus ferox* | 102.281004 | 29.878657 |
| *Corylus ferox* | 102.677049 | 30.02929 |
| *Corylus ferox* | 102.518783 | 30.097081 |
| *Corylus ferox* | 102.764848 | 30.072581 |
| *Corylus ferox* | 106.33252 | 32.39235 |
| *Corylus ferox* | 106.33172 | 32.38574 |
| *Corylus ferox* | 103.596518 | 31.483005 |
| *Corylus ferox* | 103.407973 | 31.168199 |
| *Corylus ferox* | 109.947807 | 31.443599 |
| *Corylus ferox* | 109.576403 | 31.40488 |
| *Corylus ferox* | 102.853436 | 29.799194 |
| *Corylus ferox* | 104.570975 | 28.909981 |
| *Corylus ferox* | 102.493654 | 28.617725 |
| *Corylus ferox* | 106.885477 | 31.049477 |
| *Corylus ferox* | 102.76453 | 30.072473 |
| *Corylus ferox* | 91.473 | 27.5315 |
| *Corylus ferox* | 87.772377 | 28.369911 |
| *Corylus ferox* | 87.471042 | 27.908347 |
| *Corylus ferox* | 91.96375 | 27.997004 |
| *Corylus ferox* | 85.304074 | 28.858502 |
| *Corylus ferox* | 85.98488 | 27.961912 |
| *Corylus ferox* | 90.896194 | 30.763889 |
| *Corylus ferox* | 88.913441 | 27.491109 |
| *Corylus ferox* | 95.7739 | 29.86506 |
| *Corylus ferox* | 87.424261 | 27.869925 |
| *Corylus ferox* | 86.06156 | 29.020665 |
| *Corylus ferox* | 85.989792 | 27.993572 |
| *Corylus ferox* | 91.473544 | 27.53309 |
| *Corylus ferox* | 91.473544 | 28.53309 |
| *Corylus ferox* | 85.972852 | 27.97483 |
| *Corylus ferox* | 98.15145 | 25.34116 |
| *Corylus ferox* | 103.979264 | 28.150089 |
| *Corylus ferox* | 103.983829 | 28.210213 |
| *Corylus ferox* | 103.899282 | 27.711428 |
| *Corylus ferox* | 100.31185 | 25.620941 |
| *Corylus ferox* | 98.672465 | 27.747066 |
| *Corylus ferox* | 98.672399 | 27.747068 |
| *Corylus ferox* | 98.37 | 26.1 |
| *Corylus ferox* | 102.492853 | 26.033636 |
| *Corylus ferox* | 102.1873 | 26.032746 |
| *Corylus ferox* | 103.1873 | 27.032746 |
| *Corylus ferox* | 99.495816 | 27.064386 |
| *Corylus ferox* | 102.5017 | 26.3229 |
| *Corylus ferox* | 105.5017 | 27.3229 |
| *Corylus ferox* | 106.5017 | 28.3229 |
| *Corylus ferox* | 100.419556 | 25.440268 |
| *Corylus ferox* | 102.8295 | 24.999978 |
| *Corylus ferox* | 100.29295 | 25.9322 |
| *Corylus ferox* | 98.1 | 25.33 |
| *Corylus ferox* | 99.1823 | 27.20129 |
| *Corylus ferox* | 99.053828 | 27.704502 |
| *Corylus ferox* | 99.262335 | 27.115408 |
| *Corylus ferox* | 99.6 | 27.6 |
| *Corylus ferox* | 100.6 | 28.6 |
| *Corylus ferox* | 103.064026 | 27.123558 |
| *Corylus ferox* | 103.013042 | 27.180702 |
| *Corylus ferox* | 104.054443 | 27.63169 |
| *Corylus ferox* | 104.880404 | 27.447795 |
| *Corylus ferox* | 109.113693 | 31.505398 |
| *Corylus ferox* | 109.07517 | 31.51205 |
| *Corylus ferox* | 108.30868 | 31.52137 |
| *Corylus ferox* | 108.44238 | 31.38457 |
| *Corylus ferox* | 107.105585 | 29.163479 |
| *Corylus ferox* | 107.216026 | 29.039864 |
| *Corylus ferox* | 108.319125 | 33.316796 |
| *Corylus ferox* | 98.344 | 27.373 |
| *Corylus heterophylla* | 113.334 | 35.3251 |
| *Corylus heterophylla* | 116.335109 | 40.320998 |
| *Corylus heterophylla* | 115.779219 | 40.590416 |
| *Corylus heterophylla* | 116.010818 | 40.364172 |
| *Corylus heterophylla* | 116.695387 | 40.6283 |
| *Corylus heterophylla* | 115.584772 | 39.844306 |
| *Corylus heterophylla* | 115.448072 | 39.972614 |
| *Corylus heterophylla* | 115.597528 | 39.840111 |
| *Corylus heterophylla* | 115.623196 | 39.863095 |
| *Corylus heterophylla* | 116.466056 | 40.28323 |
| *Corylus heterophylla* | 117.156401 | 40.366149 |
| *Corylus heterophylla* | 116.401862 | 40.333521 |
| *Corylus heterophylla* | 116.282055 | 40.039535 |
| *Corylus heterophylla* | 116.638673 | 40.323938 |
| *Corylus heterophylla* | 116.627057 | 40.905551 |
| *Corylus heterophylla* | 116.659205 | 40.635907 |
| *Corylus heterophylla* | 116.989011 | 40.5737 |
| *Corylus heterophylla* | 117.049035 | 40.628074 |
| *Corylus heterophylla* | 116.823045 | 40.547477 |
| *Corylus heterophylla* | 117.43804 | 40.636147 |
| *Corylus heterophylla* | 117.008135 | 40.452183 |
| *Corylus heterophylla* | 116.033186 | 40.07123 |
| *Corylus heterophylla* | 117.267571 | 40.343692 |
| *Corylus heterophylla* | 117.140653 | 40.291282 |
| *Corylus heterophylla* | 116.050093 | 40.330873 |
| *Corylus heterophylla* | 115.823054 | 40.509169 |
| *Corylus heterophylla* | 115.949938 | 39.772929 |
| *Corylus heterophylla* | 116.707695 | 40.781784 |
| *Corylus heterophylla* | 117.108992 | 40.418031 |
| *Corylus heterophylla* | 115.447577 | 39.972074 |
| *Corylus heterophylla* | 116.288499 | 39.935319 |
| *Corylus heterophylla* | 115.983072 | 40.46261 |
| *Corylus heterophylla* | 116.242115 | 40.437658 |
| *Corylus heterophylla* | 115.790552 | 40.527196 |
| *Corylus heterophylla* | 115.895804 | 40.55826 |
| *Corylus heterophylla* | 104.142071 | 35.263915 |
| *Corylus heterophylla* | 104.215148 | 35.2238 |
| *Corylus heterophylla* | 104.235173 | 35.542701 |
| *Corylus heterophylla* | 104.243779 | 35.570287 |
| *Corylus heterophylla* | 104.251945 | 35.025785 |
| *Corylus heterophylla* | 104.251945 | 35.470177 |
| *Corylus heterophylla* | 104.29447 | 35.32215 |
| *Corylus heterophylla* | 104.29447 | 35.32519 |
| *Corylus heterophylla* | 108.633406 | 35.981736 |
| *Corylus heterophylla* | 106.015432 | 34.357188 |
| *Corylus heterophylla* | 106.49235 | 35.542078 |
| *Corylus heterophylla* | 105.748478 | 33.756635 |
| *Corylus heterophylla* | 104.783703 | 32.866098 |
| *Corylus heterophylla* | 108.401834 | 36.130632 |
| *Corylus heterophylla* | 105.615392 | 33.335376 |
| *Corylus heterophylla* | 105.025195 | 34.906068 |
| *Corylus heterophylla* | 104.1428 | 33.33735 |
| *Corylus heterophylla* | 106.553128 | 34.47592 |
| *Corylus heterophylla* | 107.2227 | 26.224 |
| *Corylus heterophylla* | 105.0718 | 27.0625 |
| *Corylus heterophylla* | 106.0718 | 28.0625 |
| *Corylus heterophylla* | 105.3531 | 27.0631 |
| *Corylus heterophylla* | 106.3531 | 28.0631 |
| *Corylus heterophylla* | 106.598287 | 26.627011 |
| *Corylus heterophylla* | 106.893552 | 26.836848 |
| *Corylus heterophylla* | 103.5803 | 27.2544 |
| *Corylus heterophylla* | 106.29371 | 26.51581 |
| *Corylus heterophylla* | 107.29371 | 27.51581 |
| *Corylus heterophylla* | 104.19235 | 25.40365 |
| *Corylus heterophylla* | 105.19235 | 26.40365 |
| *Corylus heterophylla* | 106.699008 | 26.604073 |
| *Corylus heterophylla* | 104.259481 | 26.879247 |
| *Corylus heterophylla* | 106.843048 | 26.971468 |
| *Corylus heterophylla* | 105.54562 | 26.42271 |
| *Corylus heterophylla* | 106.54562 | 27.42271 |
| *Corylus heterophylla* | 117.994391 | 41.055786 |
| *Corylus heterophylla* | 118.079121 | 41.421133 |
| *Corylus heterophylla* | 118.372284 | 40.075889 |
| *Corylus heterophylla* | 117.671037 | 40.190197 |
| *Corylus heterophylla* | 117.664634 | 40.199498 |
| *Corylus heterophylla* | 117.22823 | 41.35406 |
| *Corylus heterophylla* | 119.693137 | 40.141505 |
| *Corylus heterophylla* | 119.548801 | 40.124567 |
| *Corylus heterophylla* | 114.201563 | 38.855351 |
| *Corylus heterophylla* | 117.124297 | 42.29769 |
| *Corylus heterophylla* | 116.62956 | 40.907459 |
| *Corylus heterophylla* | 117.109888 | 40.023576 |
| *Corylus heterophylla* | 114.151463 | 38.038386 |
| *Corylus heterophylla* | 115.392338 | 39.674105 |
| *Corylus heterophylla* | 114.709195 | 39.222931 |
| *Corylus heterophylla* | 115.585089 | 40.792149 |
| *Corylus heterophylla* | 117.185036 | 41.778254 |
| *Corylus heterophylla* | 114.518493 | 37.292858 |
| *Corylus heterophylla* | 114.129106 | 37.018785 |
| *Corylus heterophylla* | 118.654232 | 41.239373 |
| *Corylus heterophylla* | 113.82748 | 38.689604 |
| *Corylus heterophylla* | 118.424609 | 39.990853 |
| *Corylus heterophylla* | 118.956481 | 40.412794 |
| *Corylus heterophylla* | 118.961664 | 40.410109 |
| *Corylus heterophylla* | 118.675542 | 40.338841 |
| *Corylus heterophylla* | 119.513892 | 40.472101 |
| *Corylus heterophylla* | 114.511325 | 36.947486 |
| *Corylus heterophylla* | 116.143717 | 38.191284 |
| *Corylus heterophylla* | 114.192685 | 36.697669 |
| *Corylus heterophylla* | 114.462437 | 37.171233 |
| *Corylus heterophylla* | 117.473462 | 40.614607 |
| *Corylus heterophylla* | 115.361948 | 39.370705 |
| *Corylus heterophylla* | 118.206773 | 40.530886 |
| *Corylus heterophylla* | 115.052234 | 39.500673 |
| *Corylus heterophylla* | 117.483677 | 40.606876 |
| *Corylus heterophylla* | 115.482232 | 32.186702 |
| *Corylus heterophylla* | 111.989958 | 33.213643 |
| *Corylus heterophylla* | 112.010794 | 33.708879 |
| *Corylus heterophylla* | 112.318185 | 35.187123 |
| *Corylus heterophylla* | 110.986697 | 33.770396 |
| *Corylus heterophylla* | 112.940188 | 34.60727 |
| *Corylus heterophylla* | 112.41193 | 35.23092 |
| *Corylus heterophylla* | 112.41193 | 35.230927 |
| *Corylus heterophylla* | 110.503347 | 34.426323 |
| *Corylus heterophylla* | 111.450352 | 33.917675 |
| *Corylus heterophylla* | 112.262387 | 33.781272 |
| *Corylus heterophylla* | 111.742549 | 33.624398 |
| *Corylus heterophylla* | 111.683529 | 33.165609 |
| *Corylus heterophylla* | 113.382019 | 35.461608 |
| *Corylus heterophylla* | 113.314549 | 35.450442 |
| *Corylus heterophylla* | 113.389785 | 35.484415 |
| *Corylus heterophylla* | 113.22 | 35.27 |
| *Corylus heterophylla* | 113.23 | 35.27 |
| *Corylus heterophylla* | 112.841056 | 35.260224 |
| *Corylus heterophylla* | 112.2841 | 35.204102 |
| *Corylus heterophylla* | 120.645701 | 27.423165 |
| *Corylus heterophylla* | 129.47733 | 44.347975 |
| *Corylus heterophylla* | 124.155514 | 50.421496 |
| *Corylus heterophylla* | 128.877516 | 47.233866 |
| *Corylus heterophylla* | 123.512486 | 47.926884 |
| *Corylus heterophylla* | 126.733936 | 45.750749 |
| *Corylus heterophylla* | 129.075895 | 44.819619 |
| *Corylus heterophylla* | 132.266283 | 45.461724 |
| *Corylus heterophylla* | 132.987768 | 45.776977 |
| *Corylus heterophylla* | 131.132067 | 46.74175 |
| *Corylus heterophylla* | 131.140684 | 46.728732 |
| *Corylus heterophylla* | 130.1820515 | 45.192227 |
| *Corylus heterophylla* | 125.29907 | 48.882841 |
| *Corylus heterophylla* | 129.043308 | 47.0271 |
| *Corylus heterophylla* | 129.392127 | 46.52385 |
| *Corylus heterophylla* | 129.774496 | 47.994956 |
| *Corylus heterophylla* | 111.649443 | 32.101069 |
| *Corylus heterophylla* | 111.454712 | 31.512907 |
| *Corylus heterophylla* | 108.624769 | 30.15246 |
| *Corylus heterophylla* | 115.444078 | 31.289821 |
| *Corylus heterophylla* | 110.88507 | 31.611046 |
| *Corylus heterophylla* | 109.39546 | 31.43553 |
| *Corylus heterophylla* | 110.39546 | 32.43553 |
| *Corylus heterophylla* | 111.39546 | 33.43553 |
| *Corylus heterophylla* | 112.39546 | 34.43553 |
| *Corylus heterophylla* | 127.280118 | 42.339304 |
| *Corylus heterophylla* | 128.813774 | 42.048722 |
| *Corylus heterophylla* | 126.5047301 | 42.2225993 |
| *Corylus heterophylla* | 128.90626 | 43.118167 |
| *Corylus heterophylla* | 128.150484 | 42.463565 |
| *Corylus heterophylla* | 125.459388 | 43.808431 |
| *Corylus heterophylla* | 127.603274 | 41.955655 |
| *Corylus heterophylla* | 126.099463 | 43.318095 |
| *Corylus heterophylla* | 109.232531 | 29.804306 |
| *Corylus heterophylla* | 128.00354 | 42.22489 |
| *Corylus heterophylla* | 127.907394 | 41.622568 |
| *Corylus heterophylla* | 118.840679 | 32.057241 |
| *Corylus heterophylla* | 119.754978 | 31.294438 |
| *Corylus heterophylla* | 119.300947 | 34.659827 |
| *Corylus heterophylla* | 15.961869 | 29.530153 |
| *Corylus heterophylla* | 116.001082 | 29.561544 |
| *Corylus heterophylla* | 116.089889 | 29.66627 |
| *Corylus heterophylla* | 115.994452 | 29.552276 |
| *Corylus heterophylla* | 119.38331 | 49.30897 |
| *Corylus heterophylla* | 123.131503 | 41.030227 |
| *Corylus heterophylla* | 123.692507 | 41.492916 |
| *Corylus heterophylla* | 123.915952 | 40.885836 |
| *Corylus heterophylla* | 120.1547 | 41.5116 |
| *Corylus heterophylla* | 121.233643 | 38.808254 |
| *Corylus heterophylla* | 124.0503 | 40.2518 |
| *Corylus heterophylla* | 124.263744 | 40.983042 |
| *Corylus heterophylla* | 124.320487 | 40.64651 |
| *Corylus heterophylla* | 124.3878 | 40.6483 |
| *Corylus heterophylla* | 124.2631 | 40.5261 |
| *Corylus heterophylla* | 123.583482 | 40.726751 |
| *Corylus heterophylla* | 123.447103 | 41.807136 |
| *Corylus heterophylla* | 125.148485 | 41.434376 |
| *Corylus heterophylla* | 124.580436 | 41.23466 |
| *Corylus heterophylla* | 122.037166 | 39.085622 |
| *Corylus heterophylla* | 121.4126 | 41.3905 |
| *Corylus heterophylla* | 119.96414 | 40.957251 |
| *Corylus heterophylla* | 124.9656 | 40.6622 |
| *Corylus heterophylla* | 119.722174 | 41.91041 |
| *Corylus heterophylla* | 119.234579 | 40.647829 |
| *Corylus heterophylla* | 119.242425 | 40.732342 |
| *Corylus heterophylla* | 123.584584 | 41.724826 |
| *Corylus heterophylla* | 123.151512 | 41.037449 |
| *Corylus heterophylla* | 124.951072 | 42.53741 |
| *Corylus heterophylla* | 124.7594 | 41.3438 |
| *Corylus heterophylla* | 122.491254 | 40.061654 |
| *Corylus heterophylla* | 123.1749 | 40.2108 |
| *Corylus heterophylla* | 122.862077 | 39.929322 |
| *Corylus heterophylla* | 122.162251 | 40.181132 |
| *Corylus heterophylla* | 118.294 | 41.5025 |
| *Corylus heterophylla* | 109.205878 | 40.717513 |
| *Corylus heterophylla* | 122.21064 | 42.76881 |
| *Corylus heterophylla* | 123.729035 | 50.603368 |
| *Corylus heterophylla* | 115.729234 | 42.320142 |
| *Corylus heterophylla* | 122.044365 | 46.088464 |
| *Corylus heterophylla* | 121.642997 | 47.088385 |
| *Corylus heterophylla* | 122.0928 | 48.323209 |
| *Corylus heterophylla* | 123.451805 | 49.091028 |
| *Corylus heterophylla* | 123.26539 | 48.15469 |
| *Corylus heterophylla* | 117.785464 | 43.263275 |
| *Corylus heterophylla* | 122.771962 | 52.229219 |
| *Corylus heterophylla* | 120.034738 | 51.20692 |
| *Corylus heterophylla* | 106.393871 | 40.708669 |
| *Corylus heterophylla* | 118.44372 | 41.39116 |
| *Corylus heterophylla* | 119.35205 | 41.601542 |
| *Corylus heterophylla* | 118.464977 | 41.413402 |
| *Corylus heterophylla* | 123.211872 | 50.588356 |
| *Corylus heterophylla* | 122.175698 | 48.152698 |
| *Corylus heterophylla* | 115.622304 | 41.832504 |
| *Corylus heterophylla* | 120.691819 | 36.221298 |
| *Corylus heterophylla* | 117.96517 | 36.35477 |
| *Corylus heterophylla* | 118.433289 | 35.25704 |
| *Corylus heterophylla* | 120.142713 | 36.007528 |
| *Corylus heterophylla* | 120.635867 | 36.272549 |
| *Corylus heterophylla* | 117.110857 | 36.262014 |
| *Corylus heterophylla* | 120.391215 | 36.135958 |
| *Corylus heterophylla* | 120.357364 | 36.070634 |
| *Corylus heterophylla* | 117.026361 | 36.651235 |
| *Corylus heterophylla* | 120.608593 | 36.218024 |
| *Corylus heterophylla* | 117.094738 | 36.269893 |
| *Corylus heterophylla* | 119.202114 | 35.670254 |
| *Corylus heterophylla* | 118.289327 | 36.45152 |
| *Corylus heterophylla* | 112.11248 | 36.740263 |
| *Corylus heterophylla* | 112.158515 | 35.274113 |
| *Corylus heterophylla* | 112.966552 | 39.072728 |
| *Corylus heterophylla* | 112.115797 | 38.813217 |
| *Corylus heterophylla* | 112.497739 | 38.611084 |
| *Corylus heterophylla* | 112.703034 | 36.773556 |
| *Corylus heterophylla* | 114.10339 | 37.591986 |
| *Corylus heterophylla* | 111.269054 | 37.582493 |
| *Corylus heterophylla* | 112.709403 | 35.862566 |
| *Corylus heterophylla* | 112.807964 | 39.552573 |
| *Corylus heterophylla* | 112.02829 | 36.917705 |
| *Corylus heterophylla* | 111.205591 | 38.183237 |
| *Corylus heterophylla* | 112.094805 | 36.599282 |
| *Corylus heterophylla* | 111.929309 | 36.872011 |
| *Corylus heterophylla* | 113.28747 | 35.777938 |
| *Corylus heterophylla* | 112.326398 | 39.024342 |
| *Corylus heterophylla* | 111.22348 | 34.842925 |
| *Corylus heterophylla* | 111.239423 | 36.576694 |
| *Corylus heterophylla* | 112.193426 | 35.696364 |
| *Corylus heterophylla* | 112.00324 | 35.253742 |
| *Corylus heterophylla* | 112.025151 | 35.448026 |
| *Corylus heterophylla* | 112.550313 | 36.800731 |
| *Corylus heterophylla* | 112.344452 | 36.506103 |
| *Corylus heterophylla* | 111.22381 | 37.087676 |
| *Corylus heterophylla* | 112.571137 | 38.191374 |
| *Corylus heterophylla* | 111.920955 | 36.744929 |
| *Corylus heterophylla* | 111.663059 | 37.672637 |
| *Corylus heterophylla* | 112.471141 | 37.800349 |
| *Corylus heterophylla* | 113.227613 | 38.788943 |
| *Corylus heterophylla* | 113.321981 | 38.83858 |
| *Corylus heterophylla* | 113.724558 | 38.800767 |
| *Corylus heterophylla* | 111.16815 | 37.115287 |
| *Corylus heterophylla* | 113.119463 | 39.816659 |
| *Corylus heterophylla* | 110.853498 | 35.976442 |
| *Corylus heterophylla* | 110.853519 | 35.976331 |
| *Corylus heterophylla* | 111.114426 | 35.879558 |
| *Corylus heterophylla* | 112.679409 | 38.064621 |
| *Corylus heterophylla* | 111.725376 | 35.744429 |
| *Corylus heterophylla* | 113.928343 | 37.34021 |
| *Corylus heterophylla* | 111.490456 | 35.41794 |
| *Corylus heterophylla* | 113.777001 | 36.971541 |
| *Corylus heterophylla* | 108.998631 | 34.266054 |
| *Corylus heterophylla* | 107.048227 | 34.239386 |
| *Corylus heterophylla* | 108.587442 | 34.804777 |
| *Corylus heterophylla* | 107.087473 | 32.896421 |
| *Corylus heterophylla* | 107.5714 | 33.4049 |
| *Corylus heterophylla* | 106.83992 | 32.832526 |
| *Corylus heterophylla* | 110.083714 | 38.745049 |
| *Corylus heterophylla* | 108.018736 | 32.386163 |
| *Corylus heterophylla* | 108.458183 | 33.438369 |
| *Corylus heterophylla* | 120.609709 | 41.635082 |
| *Corylus heterophylla* | 108.492359 | 33.780159 |
| *Corylus heterophylla* | 106.243572 | 32.879531 |
| *Corylus heterophylla* | 109.97923 | 37.691427 |
| *Corylus heterophylla* | 108.539608 | 33.321332 |
| *Corylus heterophylla* | 107.669588 | 34.112193 |
| *Corylus heterophylla* | 108.772272 | 36.816961 |
| *Corylus heterophylla* | 107.028239 | 34.278442 |
| *Corylus heterophylla* | 107.325529 | 34.064248 |
| *Corylus heterophylla* | 108.3319 | 33.332 |
| *Corylus heterophylla* | 108.5238 | 34.0054 |
| *Corylus heterophylla* | 109.036301 | 34.215163 |
| *Corylus heterophylla* | 108.674108 | 35.298333 |
| *Corylus heterophylla* | 108.817617 | 35.343173 |
| *Corylus heterophylla* | 107.398981 | 33.619013 |
| *Corylus heterophylla* | 110.3014 | 38.5058 |
| *Corylus heterophylla* | 109.0252 | 35.4702 |
| *Corylus heterophylla* | 109.183679 | 33.681738 |
| *Corylus heterophylla* | 108.91358 | 34.16329 |
| *Corylus heterophylla* | 108.3695 | 33.23692 |
| *Corylus heterophylla* | 108.204706 | 33.850606 |
| *Corylus heterophylla* | 108.69627 | 33.752892 |
| *Corylus heterophylla* | 107.970764 | 31.608326 |
| *Corylus heterophylla* | 108.120414 | 30.006109 |
| *Corylus heterophylla* | 108.397229 | 30.240104 |
| *Corylus heterophylla* | 117.414579 | 40.051509 |
| *Corylus heterophylla* | 98.862846 | 28.4448 |
| *Corylus heterophylla* | 99.405393 | 25.896816 |
| *Corylus heterophylla* | 119.510156 | 119.510156 |
| *Corylus heterophylla* | 109.14767 | 31.46884 |
| *Corylus heterophylla* | 110.14767 | 32.46884 |
| *Corylus heterophylla* | 118.345005 | 29.721889 |
| *Corylus heterophylla* | 134.294107 | 48.295255 |
| *Corylus heterophylla* | 133.67575 | 45.989005 |
| *Corylus heterophylla* | 132.953622 | 46.056896 |
| *Corylus heterophylla* | 124.965921 | 48.976799 |
| *Corylus heterophylla* | 127.606579 | 45.303432 |
| *Corylus heterophylla* | 131.217334 | 46.585499 |
| *Corylus heterophylla* | 129.785572 | 46.752332 |
| *Corylus heterophylla* | 129.621441 | 46.83729 |
| *Corylus heterophylla* | 105.79694 | 32.428386 |
| *Corylus heterophylla* | 130.410555 | 48.894983 |
| *Corylus heterophylla* | 129.795101 | 46.720369 |
| *Corylus heterophylla* | 126.958628 | 49.131656 |
| *Corylus heterophylla* | 131.160717 | 46.597727 |
| *Corylus heterophylla* | 104.869226 | 33.043291 |
| *Corylus heterophylla* | 113.679835 | 24.447337 |
| *Corylus heterophylla* | 119.25287 | 40.083266 |
| *Corylus heterophylla* | 111.65708 | 33.770993 |
| *Corylus heterophylla* | 126.657488 | 45.710343 |
| *Corylus heterophylla* | 132.332116 | 45.373011 |
| *Corylus heterophylla* | 125.48287 | 43.785108 |
| *Corylus heterophylla* | 124.483691 | 42.507318 |
| *Corylus heterophylla* | 123.259864 | 42.483896 |
| *Corylus heterophylla* | 124.924375 | 42.095926 |
| *Corylus heterophylla* | 124.457945 | 42.119103 |
| *Corylus heterophylla* | 101.955849 | 36.433577 |
| *Corylus heterophylla* | 112.02193 | 35.260664 |
| *Corylus heterophylla* | 107.756398 | 34.280376 |
| *Corylus heterophylla* | 102.234705 | 29.914116 |
| *Corylus heterophylla* | 107.222713 | 26.629227 |
| *Corylus heterophylla* | 99.213299 | 27.588118 |
| *Corylus heterophylla* | 102.48 | 26.2 |
| *Corylus heterophylla* | 113.628557 | 35.731103 |
| *Corylus heterophylla* | 104.004025 | 30.6148 |
| *Corylus mandshurica* | 106.666065 | 35.225335 |
| *Corylus mandshurica* | 116.646844 | 40.308225 |
| *Corylus mandshurica* | 114.592613 | 39.846561 |
| *Corylus mandshurica* | 113.797536 | 36.954801 |
| *Corylus mandshurica* | 113.796192 | 36.970659 |
| *Corylus mandshurica* | 115.211383 | 40.385834 |
| *Corylus mandshurica* | 115.508638 | 40.045961 |
| *Corylus mandshurica* | 116.508638 | 41.045961 |
| *Corylus mandshurica* | 117.508638 | 42.045961 |
| *Corylus mandshurica* | 106.662607 | 35.221597 |
| *Corylus mandshurica* | 106.529393 | 35.169024 |
| *Corylus mandshurica* | 103.360032 | 35.433583 |
| *Corylus mandshurica* | 103.622452 | 35.236502 |
| *Corylus mandshurica* | 106.203722 | 34.331587 |
| *Corylus mandshurica* | 110.273175 | 31.431072 |
| *Corylus mandshurica* | 128.151392 | 42.340717 |
| *Corylus mandshurica* | 128.4927 | 42.174 |
| *Corylus mandshurica* | 123.0812 | 40.596 |
| *Corylus mandshurica* | 123.4514 | 40.341 |
| *Corylus mandshurica* | 123.0828 | 40.5658 |
| *Corylus mandshurica* | 109.368547 | 32.394804 |
| *Corylus mandshurica* | 108.855156 | 31.536587 |
| *Corylus mandshurica* | 114.592374 | 39.846699 |
| *Corylus mandshurica* | 126.557421 | 43.845876 |
| *Corylus mandshurica* | 115.754099 | 39.735132 |
| *Corylus mandshurica* | 116.597528 | 40.840111 |
| *Corylus mandshurica* | 117.597528 | 41.840111 |
| *Corylus mandshurica* | 118.123351 | 40.792466 |
| *Corylus mandshurica* | 115.584805 | 39.846625 |
| *Corylus mandshurica* | 115.489687 | 39.999703 |
| *Corylus mandshurica* | 115.623199 | 39.86322 |
| *Corylus mandshurica* | 115.6209 | 39.790098 |
| *Corylus mandshurica* | 115.569366 | 39.787443 |
| *Corylus mandshurica* | 116.488227 | 40.970086 |
| *Corylus mandshurica* | 116.629365 | 40.906824 |
| *Corylus mandshurica* | 115.78238 | 40.511893 |
| *Corylus mandshurica* | 116.107891 | 39.946591 |
| *Corylus mandshurica* | 117.368726 | 40.605334 |
| *Corylus mandshurica* | 116.849476 | 40.393471 |
| *Corylus mandshurica* | 116.950302 | 40.416979 |
| *Corylus mandshurica* | 117.438586 | 40.636182 |
| *Corylus mandshurica* | 116.145866 | 39.847197 |
| *Corylus mandshurica* | 117.020714 | 40.365433 |
| *Corylus mandshurica* | 115.836964 | 40.515289 |
| *Corylus mandshurica* | 116.419658 | 40.537782 |
| *Corylus mandshurica* | 104.216369 | 35.328776 |
| *Corylus mandshurica* | 103.715476 | 35.37611 |
| *Corylus mandshurica* | 102.763662 | 36.670908 |
| *Corylus mandshurica* | 102.84672 | 36.595335 |
| *Corylus mandshurica* | 103.360533 | 34.698639 |
| *Corylus mandshurica* | 104.897428 | 34.727669 |
| *Corylus mandshurica* | 103.628095 | 36.122708 |
| *Corylus mandshurica* | 104.070452 | 35.803796 |
| *Corylus mandshurica* | 114.767766 | 39.018496 |
| *Corylus mandshurica* | 117.902949 | 41.785591 |
| *Corylus mandshurica* | 115.793891 | 40.584442 |
| *Corylus mandshurica* | 115.325402 | 40.981367 |
| *Corylus mandshurica* | 114.567945 | 39.501485 |
| *Corylus mandshurica* | 117.478686 | 40.572233 |
| *Corylus mandshurica* | 117.433184 | 40.85703 |
| *Corylus mandshurica* | 118.456259 | 41.309113 |
| *Corylus mandshurica* | 119.25288 | 40.083266 |
| *Corylus mandshurica* | 119.25289 | 40.083266 |
| *Corylus mandshurica* | 119.769144 | 40.008309 |
| *Corylus mandshurica* | 114.857736 | 39.888731 |
| *Corylus mandshurica* | 115.003486 | 39.876882 |
| *Corylus mandshurica* | 117.494444 | 40.595756 |
| *Corylus mandshurica* | 117.065718 | 42.169245 |
| *Corylus mandshurica* | 117.27035 | 40.374106 |
| *Corylus mandshurica* | 122.718638 | 39.649313 |
| *Corylus mandshurica* | 131.591971 | 46.44723 |
| *Corylus mandshurica* | 124.110543 | 50.414244 |
| *Corylus mandshurica* | 129.073866 | 44.788692 |
| *Corylus mandshurica* | 130.230817 | 47.427849 |
| *Corylus mandshurica* | 128.738015 | 44.0959 |
| *Corylus mandshurica* | 128.943808 | 43.847216 |
| *Corylus mandshurica* | 128.717731 | 47.455583 |
| *Corylus mandshurica* | 130.940436 | 44.821102 |
| *Corylus mandshurica* | 129.40231 | 47.14436 |
| *Corylus mandshurica* | 128.893176 | 47.192299 |
| *Corylus mandshurica* | 126.032289 | 43.551568 |
| *Corylus mandshurica* | 127.795162 | 42.438625 |
| *Corylus mandshurica* | 127.101802 | 43.735298 |
| *Corylus mandshurica* | 128.1504 | 42.4627 |
| *Corylus mandshurica* | 126.905417 | 43.507799 |
| *Corylus mandshurica* | 131.091965 | 43.197505 |
| *Corylus mandshurica* | 126.913775 | 43.742846 |
| *Corylus mandshurica* | 129.30574 | 43.694839 |
| *Corylus mandshurica* | 128.083986 | 42.066898 |
| *Corylus mandshurica* | 124.348224 | 40.259324 |
| *Corylus mandshurica* | 123.915956 | 40.878748 |
| *Corylus mandshurica* | 124.072422 | 40.45918 |
| *Corylus mandshurica* | 123.7647 | 40.5331 |
| *Corylus mandshurica* | 124.3853 | 41.5206 |
| *Corylus mandshurica* | 122.132926 | 41.659497 |
| *Corylus mandshurica* | 125.367544 | 41.272925 |
| *Corylus mandshurica* | 124.946388 | 41.346087 |
| *Corylus mandshurica* | 124.7386 | 40.9147 |
| *Corylus mandshurica* | 124.7769 | 40.9092 |
| *Corylus mandshurica* | 124.7789 | 40.9097 |
| *Corylus mandshurica* | 124.7892 | 40.9175 |
| *Corylus mandshurica* | 124.88834 | 40.933955 |
| *Corylus mandshurica* | 124.8042 | 40.9393 |
| *Corylus mandshurica* | 123.123516 | 41.024776 |
| *Corylus mandshurica* | 124.918 | 41.9241 |
| *Corylus mandshurica* | 125.2183 | 41.965 |
| *Corylus mandshurica* | 125.1111 | 41.9033 |
| *Corylus mandshurica* | 124.6666 | 41.3637 |
| *Corylus mandshurica* | 124.5631 | 41.5325 |
| *Corylus mandshurica* | 122.4408 | 40.0508 |
| *Corylus mandshurica* | 118.463697 | 41.40995 |
| *Corylus mandshurica* | 118.369628 | 41.65564 |
| *Corylus mandshurica* | 119.350609 | 41.600245 |
| *Corylus mandshurica* | 106.1457 | 35.2231 |
| *Corylus mandshurica* | 105.735386 | 35.970076 |
| *Corylus mandshurica* | 106.261475 | 35.518691 |
| *Corylus mandshurica* | 102.83639 | 36.325561 |
| *Corylus mandshurica* | 102.495385 | 35.856667 |
| *Corylus mandshurica* | 102.665186 | 35.836454 |
| *Corylus mandshurica* | 116.972829 | 36.629637 |
| *Corylus mandshurica* | 120.623696 | 36.214561 |
| *Corylus mandshurica* | 111.556793 | 37.882106 |
| *Corylus mandshurica* | 111.447084 | 37.845795 |
| *Corylus mandshurica* | 111.4503023 | 37.8450792 |
| *Corylus mandshurica* | 111.4908 | 37.8108 |
| *Corylus mandshurica* | 111.493612 | 37.8189641 |
| *Corylus mandshurica* | 111.4937 | 37.8189 |
| *Corylus mandshurica* | 112.4503024 | 38.8450792 |
| *Corylus mandshurica* | 111.890946 | 38.685633 |
| *Corylus mandshurica* | 111.518492 | 37.796002 |
| *Corylus mandshurica* | 112.124711 | 38.930527 |
| *Corylus mandshurica* | 111.457197 | 37.854146 |
| *Corylus mandshurica* | 111.182118 | 36.98637 |
| *Corylus mandshurica* | 113.69873 | 39.711724 |
| *Corylus mandshurica* | 113.731052 | 39.667618 |
| *Corylus mandshurica* | 113.703889 | 39.567056 |
| *Corylus mandshurica* | 112.162568 | 37.557772 |
| *Corylus mandshurica* | 111.474945 | 37.833703 |
| *Corylus mandshurica* | 111.487934 | 37.880229 |
| *Corylus mandshurica* | 111.704791 | 37.766873 |
| *Corylus mandshurica* | 111.999227 | 36.895317 |
| *Corylus mandshurica* | 111.916719 | 37.027097 |
| *Corylus mandshurica* | 111.945432 | 38.365147 |
| *Corylus mandshurica* | 111.785618 | 36.853368 |
| *Corylus mandshurica* | 111.950269 | 36.808259 |
| *Corylus mandshurica* | 112.084014 | 38.837363 |
| *Corylus mandshurica* | 111.56438 | 37.491654 |
| *Corylus mandshurica* | 112.043119 | 38.803817 |
| *Corylus mandshurica* | 111.102911 | 36.41794 |
| *Corylus mandshurica* | 112.380029 | 36.524929 |
| *Corylus mandshurica* | 112.318269 | 38.102859 |
| *Corylus mandshurica* | 111.922879 | 36.733747 |
| *Corylus mandshurica* | 112.09909 | 36.631252 |
| *Corylus mandshurica* | 113.261451 | 38.734182 |
| *Corylus mandshurica* | 113.426759 | 38.569411 |
| *Corylus mandshurica* | 111.85341 | 38.91702 |
| *Corylus mandshurica* | 112.827315 | 36.924429 |
| *Corylus mandshurica* | 111.294434 | 37.144161 |
| *Corylus mandshurica* | 113.595708 | 38.976198 |
| *Corylus mandshurica* | 111.866577 | 38.739115 |
| *Corylus mandshurica* | 112.9413 | 38.398047 |
| *Corylus mandshurica* | 106.683665 | 34.721433 |
| *Corylus mandshurica* | 110.073028 | 34.497647 |
| *Corylus mandshurica* | 109.17864 | 32.59639 |
| *Corylus mandshurica* | 109.17864 | 32.259639 |
| *Corylus mandshurica* | 110.17864 | 33.59639 |
| *Corylus mandshurica* | 113.16576 | 36.0214 |
| *Corylus mandshurica* | 114.16576 | 37.0214 |
| *Corylus mandshurica* | 108.738596 | 35.900295 |
| *Corylus mandshurica* | 102.132207 | 29.648553 |
| *Corylus mandshurica* | 102.974391 | 33.584377 |
| *Corylus mandshurica* | 104.342607 | 29.940476 |
| *Corylus mandshurica* | 107.883382 | 29.718506 |
| *Corylus mandshurica* | 127.194246 | 45.431056 |
| *Corylus mandshurica* | 127.968219 | 45.219455 |
| *Corylus mandshurica* | 117.47949 | 40.351909 |
| *Corylus mandshurica* | 133.505217 | 46.699092 |
| *Corylus mandshurica* | 126.692904 | 46.827103 |
| *Corylus mandshurica* | 128.091594 | 42.059454 |
| *Corylus mandshurica* | 113.820849 | 38.683939 |
| *Corylus mandshurica* | 128.009679 | 45.214499 |
| *Corylus mandshurica* | 128.195573 | 43.975443 |
| *Corylus mandshurica* | 118.536541 | 41.65052 |
| *Corylus heterophylla* var*. sutchuanensis* | 106.094418 | 33.775194 |
| *Corylus heterophylla* var*. sutchuanensis* | 115.554802 | 29.314224 |
| *Corylus heterophylla* var*. sutchuanensis* | 119.438938 | 30.41837 |
| *Corylus heterophylla* var*. sutchuanensis* | 119.379976 | 28.451428 |
| *Corylus heterophylla* var*. sutchuanensis* | 121.103742 | 29.254121 |
| *Corylus heterophylla* var*. sutchuanensis* | 121.098923 | 29.257378 |
| *Corylus heterophylla* var*. sutchuanensis* | 120.5698 | 29.006182 |
| *Corylus heterophylla* var*. sutchuanensis* | 121.0997 | 29.258215 |
| *Corylus heterophylla* var*. sutchuanensis* | 106.704435 | 26.540042 |
| *Corylus heterophylla* var*. sutchuanensis* | 110.079559 | 28.668713 |
| *Corylus heterophylla* var*. sutchuanensis* | 107.821651 | 34.061745 |
| *Corylus heterophylla* var*. sutchuanensis* | 102.606948 | 31.527342 |
| *Corylus heterophylla* var*. sutchuanensis* | 106.34238 | 26.54451 |
| *Corylus heterophylla* var*. sutchuanensis* | 107.34238 | 27.54451 |
| *Corylus heterophylla* var*. sutchuanensis* | 107.340017 | 28.424615 |
| *Corylus heterophylla* var*. sutchuanensis* | 105.5258 | 26.5311 |
| *Corylus heterophylla* var*. sutchuanensis* | 106.5258 | 27.5311 |
| *Corylus heterophylla* var*. sutchuanensis* | 110.557883 | 34.536105 |
| *Corylus heterophylla* var*. sutchuanensis* | 115.482195 | 31.763022 |
| *Corylus heterophylla* var*. sutchuanensis* | 115.043985 | 32.480985 |
| *Corylus heterophylla* var*. sutchuanensis* | 114.3428 | 29.2322 |
| *Corylus heterophylla* var*. sutchuanensis* | 114.3827 | 29.2317 |
| *Corylus heterophylla* var*. sutchuanensis* | 114.3727 | 29.2302 |
| *Corylus heterophylla* var*. sutchuanensis* | 119.45208 | 34.711685 |
| *Corylus heterophylla* var*. sutchuanensis* | 115.5653 | 29.3132 |
| *Corylus heterophylla* var*. sutchuanensis* | 116.5653 | 30.3132 |
| *Corylus heterophylla* var*. sutchuanensis* | 117.5653 | 31.3132 |
| *Corylus heterophylla* var*. sutchuanensis* | 118.5653 | 32.3132 |
| *Corylus heterophylla* var*. sutchuanensis* | 119.5653 | 33.3132 |
| *Corylus heterophylla* var*. sutchuanensis* | 116.005 | 29.3337 |
| *Corylus heterophylla* var*. sutchuanensis* | 115.5737 | 29.3309 |
| *Corylus heterophylla* var*. sutchuanensis* | 109.159575 | 33.429045 |
| *Corylus heterophylla* var*. sutchuanensis* | 108.307497 | 33.90622 |
| *Corylus heterophylla* var*. sutchuanensis* | 101.964544 | 30.001969 |
| *Corylus heterophylla* var*. sutchuanensis* | 103.551585 | 31.798511 |
| *Corylus heterophylla* var*. sutchuanensis* | 109.985929 | 31.407203 |
| *Corylus heterophylla* var*. sutchuanensis* | 121.013553 | 29.149514 |
| *Corylus heterophylla* var*. sutchuanensis* | 107.224829 | 29.053724 |
| *Corylus heterophylla* var*. sutchuanensis* | 111.327898 | 29.429739 |
| *Corylus heterophylla* var*. sutchuanensis* | 109.849941 | 29.703798 |
| *Corylus heterophylla* var*. sutchuanensis* | 105.986604 | 26.21181 |
| *Corylus heterophylla* var*. sutchuanensis* | 115.987852 | 29.546589 |
| *Corylus heterophylla* var*. sutchuanensis* | 117.359435 | 30.197506 |
| *Corylus heterophylla* var*. sutchuanensis* | 118.168089 | 30.13039 |
| *Corylus heterophylla* var*. sutchuanensis* | 118.17822 | 30.148366 |
| *Corylus heterophylla* var*. sutchuanensis* | 118.194596 | 30.094975 |
| *Corylus heterophylla* var*. sutchuanensis* | 117.830511 | 30.484894 |
| *Corylus heterophylla* var*. sutchuanensis* | 118.182231 | 30.128638 |
| *Corylus heterophylla* var*. sutchuanensis* | 116.366555 | 30.855228 |
| *Corylus heterophylla* var*. sutchuanensis* | 105.612556 | 33.333454 |
| *Corylus heterophylla* var*. sutchuanensis* | 104.828293 | 32.796543 |
| *Corylus heterophylla* var*. sutchuanensis* | 104.366741 | 33.571401 |
| *Corylus heterophylla* var*. sutchuanensis* | 106.906183 | 26.877885 |
| *Corylus heterophylla* var*. sutchuanensis* | 105.449543 | 25.10399 |
| *Corylus heterophylla* var*. sutchuanensis* | 105.17309 | 26.15345 |
| *Corylus heterophylla* var*. sutchuanensis* | 106.17309 | 27.15345 |
| *Corylus heterophylla* var*. sutchuanensis* | 106.176836 | 25.58734 |
| *Corylus heterophylla* var*. sutchuanensis* | 105.827507 | 28.429532 |
| *Corylus heterophylla* var*. sutchuanensis* | 107.614053 | 29.157899 |
| *Corylus heterophylla* var*. sutchuanensis* | 107.619484 | 28.86865 |
| *Corylus heterophylla* var*. sutchuanensis* | 106.143721 | 26.12268 |
| *Corylus heterophylla* var*. sutchuanensis* | 107.143721 | 27.12268 |
| *Corylus heterophylla* var*. sutchuanensis* | 106.658165 | 26.384425 |
| *Corylus heterophylla* var*. sutchuanensis* | 104.710323 | 27.118776 |
| *Corylus heterophylla* var*. sutchuanensis* | 104.898552 | 27.046548 |
| *Corylus heterophylla* var*. sutchuanensis* | 106.97156 | 27.063674 |
| *Corylus heterophylla* var*. sutchuanensis* | 107.893409 | 25.417098 |
| *Corylus heterophylla* var*. sutchuanensis* | 105.471396 | 26.232343 |
| *Corylus heterophylla* var*. sutchuanensis* | 106.573967 | 26.374363 |
| *Corylus heterophylla* var*. sutchuanensis* | 107.573967 | 27.374363 |
| *Corylus heterophylla* var*. sutchuanensis* | 108.573967 | 28.374363 |
| *Corylus heterophylla* var*. sutchuanensis* | 105.310692 | 25.250137 |
| *Corylus heterophylla* var*. sutchuanensis* | 106.830729 | 27.2118 |
| *Corylus heterophylla* var*. sutchuanensis* | 108.510478 | 28.570102 |
| *Corylus heterophylla* var*. sutchuanensis* | 108.215992 | 28.713516 |
| *Corylus heterophylla* var*. sutchuanensis* | 105.5426 | 28.2144 |
| *Corylus heterophylla* var*. sutchuanensis* | 107.702012 | 26.121603 |
| *Corylus heterophylla* var*. sutchuanensis* | 106.869249 | 27.949819 |
| *Corylus heterophylla* var*. sutchuanensis* | 111.64083 | 33.723215 |
| *Corylus heterophylla* var*. sutchuanensis* | 112.914434 | 33.744879 |
| *Corylus heterophylla* var*. sutchuanensis* | 111.659587 | 34.394985 |
| *Corylus heterophylla* var*. sutchuanensis* | 115.413513 | 31.804345 |
| *Corylus heterophylla* var*. sutchuanensis* | 111.824079 | 33.641367 |
| *Corylus heterophylla* var*. sutchuanensis* | 114.885525 | 31.649738 |
| *Corylus heterophylla* var*. sutchuanensis* | 114.043985 | 31.480985 |
| *Corylus heterophylla* var*. sutchuanensis* | 112.005709 | 34.511671 |
| *Corylus heterophylla* var*. sutchuanensis* | 114.890224 | 33.053458 |
| *Corylus heterophylla* var*. sutchuanensis* | 115.054665 | 31.596747 |
| *Corylus heterophylla* var*. sutchuanensis* | 110.2042 | 51.487586 |
| *Corylus heterophylla* var*. sutchuanensis* | 116.010733 | 30.580962 |
| *Corylus heterophylla* var*. sutchuanensis* | 110.508799 | 31.801078 |
| *Corylus heterophylla* var*. sutchuanensis* | 110.753446 | 31.354464 |
| *Corylus heterophylla* var*. sutchuanensis* | 110.904678 | 31.160037 |
| *Corylus heterophylla* var*. sutchuanensis* | 110.93614 | 31.066945 |
| *Corylus heterophylla* var*. sutchuanensis* | 110.647785 | 31.357935 |
| *Corylus heterophylla* var*. sutchuanensis* | 28.718611 | 28.718611 |
| *Corylus heterophylla* var*. sutchuanensis* | 112.722068 | 27.27567 |
| *Corylus heterophylla* var*. sutchuanensis* | 109.155506 | 28.581991 |
| *Corylus heterophylla* var*. sutchuanensis* | 111.309125 | 28.154757 |
| *Corylus heterophylla* var*. sutchuanensis* | 111.09713 | 26.606407 |
| *Corylus heterophylla* var*. sutchuanensis* | 111.464449 | 27.327132 |
| *Corylus heterophylla* var*. sutchuanensis* | 110.208485 | 28.738161 |
| *Corylus heterophylla* var*. sutchuanensis* | 110.491514 | 29.052912 |
| *Corylus heterophylla* var*. sutchuanensis* | 115.922198 | 29.411011 |
| *Corylus heterophylla* var*. sutchuanensis* | 115.974762 | 29.556365 |
| *Corylus heterophylla* var*. sutchuanensis* | 114.424173 | 28.939113 |
| *Corylus heterophylla* var*. sutchuanensis* | 115.968465 | 29.529537 |
| *Corylus heterophylla* var*. sutchuanensis* | 115.935867 | 29.536433 |
| *Corylus heterophylla* var*. sutchuanensis* | 115.938201 | 29.53733 |
| *Corylus heterophylla* var*. sutchuanensis* | 115.944402 | 29.533291 |
| *Corylus heterophylla* var*. sutchuanensis* | 115.971486 | 29.555959 |
| *Corylus heterophylla* var*. sutchuanensis* | 115.960859 | 29.54856 |
| *Corylus heterophylla* var*. sutchuanensis* | 115.972575 | 29.557887 |
| *Corylus heterophylla* var*. sutchuanensis* | 114.883086 | 29.402223 |
| *Corylus heterophylla* var*. sutchuanensis* | 115.22442 | 27.58718 |
| *Corylus heterophylla* var*. sutchuanensis* | 114.553568 | 29.031176 |
| *Corylus heterophylla* var*. sutchuanensis* | 114.553156 | 29.031876 |
| *Corylus heterophylla* var*. sutchuanensis* | 118.229842 | 28.819943 |
| *Corylus heterophylla* var*. sutchuanensis* | 109.303175 | 37.427398 |
| *Corylus heterophylla* var*. sutchuanensis* | 105.938588 | 33.358364 |
| *Corylus heterophylla* var*. sutchuanensis* | 110.4843 | 33.14904 |
| *Corylus heterophylla* var*. sutchuanensis* | 111.4843 | 34.14904 |
| *Corylus heterophylla* var*. sutchuanensis* | 112.4843 | 35.14904 |
| *Corylus heterophylla* var*. sutchuanensis* | 113.4843 | 36.14904 |
| *Corylus heterophylla* var*. sutchuanensis* | 106.909991 | 34.237509 |
| *Corylus heterophylla* var*. sutchuanensis* | 107.997424 | 33.530693 |
| *Corylus heterophylla* var*. sutchuanensis* | 108.531852 | 33.828983 |
| *Corylus heterophylla* var*. sutchuanensis* | 108.254427 | 33.044602 |
| *Corylus heterophylla* var*. sutchuanensis* | 109.259175 | 35.023824 |
| *Corylus heterophylla* var*. sutchuanensis* | 104.407314 | 35.846827 |
| *Corylus heterophylla* var*. sutchuanensis* | 109.2312 | 31.57956 |
| *Corylus heterophylla* var*. sutchuanensis* | 110.2312 | 32.57956 |
| *Corylus heterophylla* var*. sutchuanensis* | 111.2312 | 33.57956 |
| *Corylus heterophylla* var*. sutchuanensis* | 109.23777 | 31.57119 |
| *Corylus heterophylla* var*. sutchuanensis* | 110.23777 | 32.57119 |
| *Corylus heterophylla* var*. sutchuanensis* | 111.23777 | 33.57119 |
| *Corylus heterophylla* var*. sutchuanensis* | 112.23777 | 34.57119 |
| *Corylus heterophylla* var*. sutchuanensis* | 113.23777 | 35.57119 |
| *Corylus heterophylla* var*. sutchuanensis* | 104.361586 | 31.963084 |
| *Corylus heterophylla* var*. sutchuanensis* | 103.85959 | 31.686809 |
| *Corylus heterophylla* var*. sutchuanensis* | 108.402076 | 30.246057 |
| *Corylus heterophylla* var*. sutchuanensis* | 107.33252 | 33.39235 |
| *Corylus heterophylla* var*. sutchuanensis* | 119.510156 | 30.345729 |
| *Corylus heterophylla* var*. sutchuanensis* | 119.443919 | 30.412799 |
| *Corylus heterophylla* var*. sutchuanensis* | 119.224435 | 30.168569 |
| *Corylus heterophylla* var*. sutchuanensis* | 120.694334 | 27.760385 |
| *Corylus heterophylla* var*. sutchuanensis* | 119.455097 | 30.340834 |
| *Corylus heterophylla* var*. sutchuanensis* | 119.46893 | 30.175207 |
| *Corylus heterophylla* var*. sutchuanensis* | 119.485966 | 30.338041 |
| *Corylus heterophylla* var*. sutchuanensis* | 119.454742 | 30.317643 |
| *Corylus heterophylla* var*. sutchuanensis* | 121.04204 | 29.162747 |
| *Corylus heterophylla* var*. sutchuanensis* | 108.59534 | 31.752408 |
| *Corylus heterophylla* var*. sutchuanensis* | 108.287703 | 31.96715 |
| *Corylus heterophylla* var*. sutchuanensis* | 108.2651 | 30.153 |
| *Corylus heterophylla* var*. sutchuanensis* | 106.957384 | 26.896508 |
| *Corylus heterophylla* var*. sutchuanensis* | 114.965833 | 39.950222 |
| *Corylus heterophylla* var*. sutchuanensis* | 119.436458 | 30.353794 |
| *Corylus heterophylla* var*. sutchuanensis* | 111.386451 | 29.590366 |
| *Corylus heterophylla* var*. sutchuanensis* | 118.823369 | 32.085309 |
| *Corylus heterophylla* var*. sutchuanensis* | 105.1548 | 25.18398 |
| *Corylus heterophylla* var*. sutchuanensis* | 106.1548 | 26.18398 |
| *Corylus heterophylla* var*. sutchuanensis* | 107.1548 | 27.18398 |
| *Corylus heterophylla* var*. sutchuanensis* | 108.1548 | 28.18398 |
| *Corylus heterophylla* var*. sutchuanensis* | 107.731 | 29.863399 |
| *Corylus heterophylla* var*. sutchuanensis* | 106.187535 | 27.851364 |
| *Corylus heterophylla* var*. sutchuanensis* | 120.277982 | 29.443079 |
| *Corylus heterophylla* var*. sutchuanensis* | 121.007637 | 29.159387 |
| *Corylus heterophylla* var*. sutchuanensis* | 116.198169 | 31.130916 |
| *Corylus heterophylla* var*. sutchuanensis* | 107.696223 | 26.053392 |
| *Corylus heterophylla* var*. sutchuanensis* | 115.983762 | 29.56479 |
| *Corylus heterophylla* var*. sutchuanensis* | 115.952542 | 29.546039 |
| *Corylus heterophylla* var*. sutchuanensis* | 116.015609 | 29.551024 |
| *Corylus heterophylla* var*. sutchuanensis* | 115.953791 | 29.485664 |
| *Corylus ferox* var*. thibetica* | 110.613977 | 31.283082 |
| *Corylus ferox* var*. thibetica* | 107.504691 | 34.003762 |
| *Corylus ferox* var*. thibetica* | 108.948317 | 31.745329 |
| *Corylus ferox* var*. thibetica* | 103.559887 | 31.573085 |
| *Corylus ferox* var*. thibetica* | 103.718851 | 30.244487 |
| *Corylus ferox* var*. thibetica* | 103.964552 | 28.604278 |
| *Corylus ferox* var*. thibetica* | 102.49359 | 26.0335 |
| *Corylus ferox* var*. thibetica* | 103.379602 | 29.910616 |
| *Corylus ferox* var*. thibetica* | 108.700569 | 27.919654 |
| *Corylus ferox* var*. thibetica* | 104.312403 | 30.568211 |
| *Corylus ferox* var*. thibetica* | 102.757549 | 29.329579 |
| *Corylus ferox* var*. thibetica* | 110.34834 | 30.81418 |
| *Corylus ferox* var*. thibetica* | 106.454304 | 29.517362 |
| *Corylus ferox* var*. thibetica* | 102.366405 | 29.234305 |
| *Corylus ferox* var*. thibetica* | 104.116798 | 30.427701 |
| *Corylus ferox* var*. thibetica* | 106.005231 | 27.55145 |
| *Corylus ferox* var*. thibetica* | 104.691794 | 26.642625 |
| *Corylus ferox* var*. thibetica* | 110.347575 | 31.048064 |
| *Corylus ferox* var*. thibetica* | 111.268594 | 31.884338 |
| *Corylus ferox* var*. thibetica* | 109.829293 | 30.145896 |
| *Corylus ferox* var*. thibetica* | 110.682525 | 31.750496 |
| *Corylus ferox* var*. thibetica* | 110.483944 | 31.537554 |
| *Corylus ferox* var*. thibetica* | 109.871165 | 29.654372 |
| *Corylus ferox* var*. thibetica* | 110.545256 | 29.679508 |
| *Corylus ferox* var*. thibetica* | 110.102327 | 29.789602 |
| *Corylus ferox* var*. thibetica* | 110.676654 | 30.009462 |
| *Corylus ferox* var*. thibetica* | 106.163544 | 33.333194 |
| *Corylus ferox* var*. thibetica* | 109.011587 | 34.371708 |
| *Corylus ferox* var*. thibetica* | 108.960386 | 34.257952 |
| *Corylus ferox* var*. thibetica* | 109.4843 | 32.14904 |
| *Corylus ferox* var*. thibetica* | 117.4843 | 40.14904 |
| *Corylus ferox* var*. thibetica* | 118.4843 | 41.14904 |
| *Corylus ferox* var*. thibetica* | 119.4843 | 42.14904 |
| *Corylus ferox* var*. thibetica* | 120.4843 | 43.14904 |
| *Corylus ferox* var*. thibetica* | 108.5035 | 33.3314 |
| *Corylus ferox* var*. thibetica* | 108.50351 | 33.3314 |
| *Corylus ferox* var*. thibetica* | 108.50352 | 33.3314 |
| *Corylus ferox* var*. thibetica* | 108.3033 | 33.2827 |
| *Corylus ferox* var*. thibetica* | 108.320564 | 33.316555 |
| *Corylus ferox* var*. thibetica* | 109.16576 | 32.0214 |
| *Corylus ferox* var*. thibetica* | 110.16576 | 33.0214 |
| *Corylus ferox* var*. thibetica* | 111.16576 | 34.0214 |
| *Corylus ferox* var*. thibetica* | 112.16576 | 35.0214 |
| *Corylus ferox* var*. thibetica* | 108.558235 | 34.041877 |
| *Corylus ferox* var*. thibetica* | 104.683831 | 31.049244 |
| *Corylus ferox* var*. thibetica* | 103.554995 | 30.961146 |
| *Corylus ferox* var*. thibetica* | 109.470473 | 31.024602 |
| *Corylus ferox* var*. thibetica* | 109.594525 | 30.725764 |
| *Corylus ferox* var*. thibetica* | 103.254549 | 27.702145 |
| *Corylus ferox* var*. thibetica* | 106.382787 | 32.498391 |
| *Corylus ferox* var*. thibetica* | 103.578429 | 28.268951 |
| *Corylus ferox* var*. thibetica* | 107.381973 | 28.987777 |
| *Corylus ferox* var*. thibetica* | 105.876507 | 28.859645 |
| *Corylus ferox* var*. thibetica* | 107.134485 | 29.055362 |
| *Corylus ferox* var*. thibetica* | 106.834486 | 32.354484 |
| *Corylus ferox* var*. thibetica* | 106.834483 | 32.352532 |
| *Corylus ferox* var*. thibetica* | 104.535422 | 32.414682 |
| *Corylus ferox* var*. thibetica* | 108.234414 | 30.268797 |
| *Corylus ferox* var*. thibetica* | 107.527785 | 32.141866 |
| *Corylus ferox* var*. thibetica* | 104.553222 | 28.705078 |
| *Corylus ferox* var*. thibetica* | 107.251517 | 31.917714 |
| *Corylus ferox* var*. thibetica* | 109.885546 | 31.080519 |
| *Corylus ferox* var*. thibetica* | 109.08992 | 31.549584 |
| *Corylus ferox* var*. thibetica* | 102.973258 | 29.776839 |
| *Corylus ferox* var*. thibetica* | 107.756246 | 29.330996 |
| *Corylus ferox* var*. thibetica* | 107.55111 | 29.399898 |
| *Corylus ferox* var*. thibetica* | 104.1942 | 32.5348 |
| *Corylus ferox* var*. thibetica* | 104.5183 | 33.5451 |
| *Corylus ferox* var*. thibetica* | 105.646793 | 33.24825 |
| *Corylus ferox* var*. thibetica* | 105.445597 | 33.433056 |
| *Corylus ferox* var*. thibetica* | 106.518916 | 34.328135 |
| *Corylus ferox* var*. thibetica* | 104.465955 | 32.483793 |
| *Corylus ferox* var*. thibetica* | 105.877205 | 32.980362 |
| *Corylus ferox* var*. thibetica* | 105.230932 | 32.684926 |
| *Corylus ferox* var*. thibetica* | 104.19187 | 32.55209 |
| *Corylus ferox* var*. thibetica* | 104.460764 | 32.994608 |
| *Corylus ferox* var*. thibetica* | 104.1173 | 33.402 |
| *Corylus ferox* var*. thibetica* | 106.221881 | 26.623901 |
| *Corylus ferox* var*. thibetica* | 104.130459 | 26.747509 |
| *Corylus ferox* var*. thibetica* | 111.512238 | 32.07727 |
| *Corylus ferox* var*. thibetica* | 110.391757 | 31.443575 |
| *Corylus ferox* var*. thibetica* | 110.243293 | 31.627653 |
| *Corylus ferox* var*. thibetica* | 110.312159 | 31.615427 |
| *Corylus ferox* var*. thibetica* | 109.835791 | 29.705595 |
| *Corylus ferox* var*. thibetica* | 108.482587 | 33.857671 |
| *Corylus ferox* var*. thibetica* | 106.59539 | 32.40595 |
| *Corylus ferox* var*. thibetica* | 91.963748 | 27.997005 |
| *Corylus ferox* var*. thibetica* | 109.622049 | 31.40151 |
| *Corylus ferox* var*. thibetica* | 110.401715 | 31.393673 |
| *Corylus ferox* var*. thibetica* | 105.49274 | 33.382794 |
| *Corylus ferox* var*. thibetica* | 108.709918 | 33.292716 |
| *Corylus ferox* var*. thibetica* | 109.19942 | 31.1408 |
| *Corylus ferox* var*. thibetica* | 34.077492 | 33.335376 |
| *Corylus ferox* var*. thibetica* | 111.429636 | 34.1408 |
| *Corylus ferox* var*. thibetica* | 112.246242 | 37.948054 |
| *Corylus ferox* var*. thibetica* | 106.461948 | 29.584126 |
| *Corylus ferox* var*. thibetica* | 105.8018 | 29.992345 |
| *Corylus ferox* var*. thibetica* | 109.806029 | 29.569498 |
| *Corylus yunnanensis* | 100.220684 | 26.865726 |
| *Corylus yunnanensis* | 102.667263 | 27.606112 |
| *Corylus yunnanensis* | 103.010957 | 30.005386 |
| *Corylus yunnanensis* | 105.38157 | 27.418584 |
| *Corylus yunnanensis* | 104.442639 | 26.844397 |
| *Corylus yunnanensis* | 104.547245 | 26.648396 |
| *Corylus yunnanensis* | 104.026275 | 26.747919 |
| *Corylus yunnanensis* | 103.96651 | 26.590501 |
| *Corylus yunnanensis* | 103.542361 | 26.312284 |
| *Corylus yunnanensis* | 104.542361 | 27.312284 |
| *Corylus yunnanensis* | 106.725176 | 26.838543 |
| *Corylus yunnanensis* | 109.983048 | 31.48854 |
| *Corylus yunnanensis* | 110.29831 | 31.192349 |
| *Corylus yunnanensis* | 111.213526 | 31.298933 |
| *Corylus yunnanensis* | 108.394418 | 33.396392 |
| *Corylus yunnanensis* | 102.895786 | 27.576395 |
| *Corylus yunnanensis* | 101.572852 | 30.0244 |
| *Corylus yunnanensis* | 102.572852 | 31.0244 |
| *Corylus yunnanensis* | 102.125327 | 30.103185 |
| *Corylus yunnanensis* | 102.658576 | 29.350217 |
| *Corylus yunnanensis* | 102.028749 | 102.028749 |
| *Corylus yunnanensis* | 101.513416 | 29.006432 |
| *Corylus yunnanensis* | 105.377727 | 28.868568 |
| *Corylus yunnanensis* | 101.958942 | 30.004793 |
| *Corylus yunnanensis* | 101.978292 | 30.05082 |
| *Corylus yunnanensis* | 102.132243 | 27.276948 |
| *Corylus yunnanensis* | 104.052252 | 30.379292 |
| *Corylus yunnanensis* | 107.747969 | 27.310962 |
| *Corylus yunnanensis* | 102.834627 | 27.216991 |
| *Corylus yunnanensis* | 103.4812 | 31.3718 |
| *Corylus yunnanensis* | 101.286397 | 27.934599 |
| *Corylus yunnanensis* | 101.150539 | 27.88261 |
| *Corylus yunnanensis* | 100.849815 | 28.150498 |
| *Corylus yunnanensis* | 101.284743 | 27.935753 |
| *Corylus yunnanensis* | 103.795818 | 30.148597 |
| *Corylus yunnanensis* | 104.387452 | 31.477098 |
| *Corylus yunnanensis* | 104.817711 | 30.456494 |
| *Corylus yunnanensis* | 103.596635 | 31.482742 |
| *Corylus yunnanensis* | 103.591805 | 31.484001 |
| *Corylus yunnanensis* | 102.278842 | 27.842075 |
| *Corylus yunnanensis* | 103.124403 | 30.020956 |
| *Corylus yunnanensis* | 102.394779 | 24.950394 |
| *Corylus yunnanensis* | 102.488744 | 24.50882 |
| *Corylus yunnanensis* | 105.179288 | 27.581262 |
| *Corylus yunnanensis* | 101.684822 | 25.051125 |
| *Corylus yunnanensis* | 100.31346 | 25.233439 |
| *Corylus yunnanensis* | 98.924617 | 28.469843 |
| *Corylus yunnanensis* | 99.563915 | 25.988475 |
| *Corylus yunnanensis* | 102.505565 | 25.224036 |
| *Corylus yunnanensis* | 104.581423 | 25.161467 |
| *Corylus yunnanensis* | 100.18223 | 26.47145 |
| *Corylus yunnanensis* | 99.514185 | 25.851206 |
| *Corylus yunnanensis* | 102.49478 | 25.45252 |
| *Corylus yunnanensis* | 102.974637 | 25.431776 |
| *Corylus yunnanensis* | 102.766042 | 25.095962 |
| *Corylus yunnanensis* | 102.4947 | 25.1358 |
| *Corylus yunnanensis* | 102.630717 | 25.071809 |
| *Corylus yunnanensis* | 104.703891 | 27.72902 |
| *Corylus yunnanensis* | 100.651866 | 26.459894 |
| *Corylus yunnanensis* | 100.202838 | 26.99827 |
| *Corylus yunnanensis* | 103.330054 | 24.817625 |
| *Corylus yunnanensis* | 102.477423 | 25.557672 |
| *Corylus yunnanensis* | 102.625062 | 25.869982 |
| *Corylus yunnanensis* | 102.269696 | 26.159198 |
| *Corylus yunnanensis* | 103.268839 | 24.805896 |
| *Corylus yunnanensis* | 103.950426 | 25.904592 |
| *Corylus yunnanensis* | 104.039533 | 24.837008 |
| *Corylus yunnanensis* | 103.35282 | 24.38242 |
| *Corylus yunnanensis* | 104.35282 | 25.38242 |
| *Corylus yunnanensis* | 105.35282 | 26.38242 |
| *Corylus yunnanensis* | 106.35282 | 27.38242 |
| *Corylus yunnanensis* | 107.35282 | 28.38242 |
| *Corylus yunnanensis* | 108.35282 | 29.38242 |
| *Corylus yunnanensis* | 102.506181 | 23.725767 |
| *Corylus yunnanensis* | 102.955323 | 25.290453 |
| *Corylus yunnanensis* | 103.2 | 25.252 |
| *Corylus yunnanensis* | 100.06447 | 26.282057 |
| *Corylus yunnanensis* | 98.467577 | 25.445325 |
| *Corylus yunnanensis* | 98.28 | 25.26 |
| *Corylus yunnanensis* | 102.570554 | 24.813511 |
| *Corylus yunnanensis* | 99.362207 | 27.33198 |
| *Corylus yunnanensis* | 102.443885 | 24.90518 |
| *Corylus yunnanensis* | 99.970914 | 25.674929 |
| *Corylus yunnanensis* | 100.333661 | 26.40392 |
| *Corylus yunnanensis* | 100.221116 | 27.0201 |
| *Corylus yunnanensis* | 103.055982 | 27.094698 |
| *Corylus yunnanensis* | 104.055982 | 28.094698 |
| *Corylus yunnanensis* | 100.06073 | 25.46531 |
| *Corylus yunnanensis* | 101.897091 | 28.684435 |
