# Supplementary figures and images for: Predicting the Impact of Climate Change on Corylus Species Distribution in China: Integrating Climatic, Topographic, and Anthropogenic Factors
Source: Ecol Evol. 2024 Nov 3;14(11):e70528. doi: 10.1002/ece3.70528 (PMC11532234; doi:10.1002/ece3.70528)

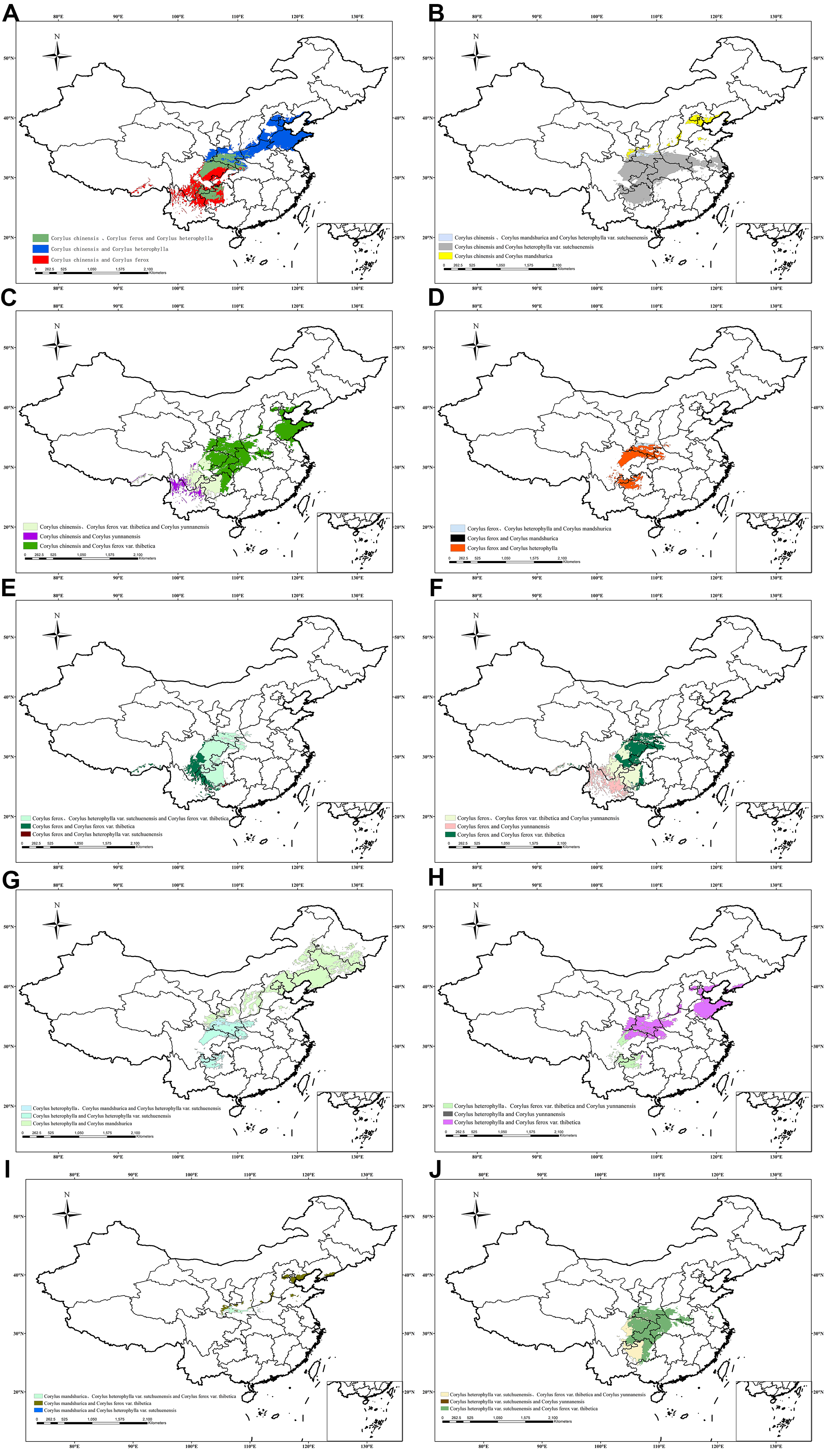

Supplement: Supplementary file 1 — Appendix S1. [file ECE3-14-e70528-s001.zip › Fig. S3.tif]

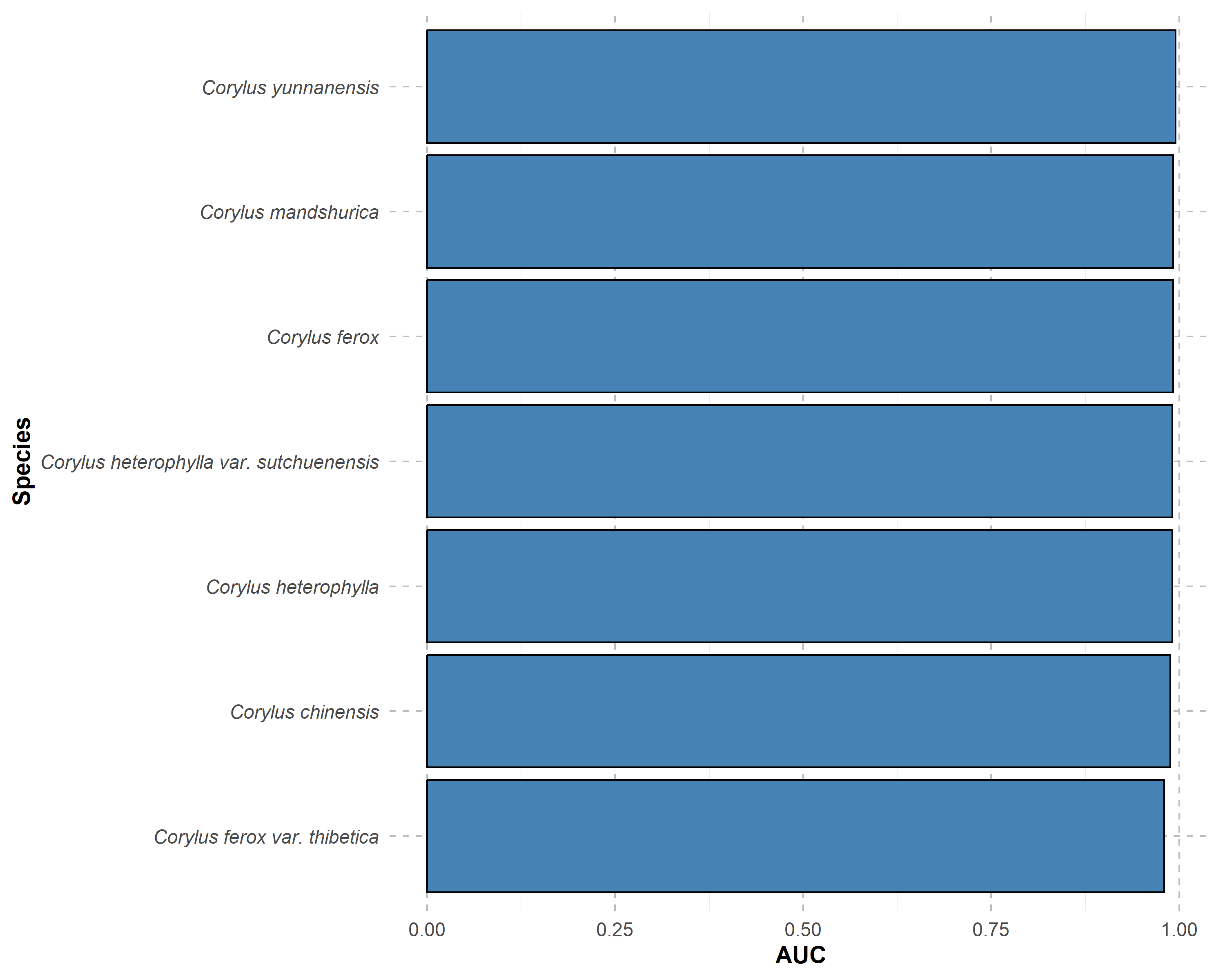

Supplement: Supplementary file 1 — Appendix S1. [file ECE3-14-e70528-s001.zip › Fig. S1.tif]
